# Meta-Analysis Reveals Transcription Factor Upregulation in Cells of Injured Mouse Sciatic Nerve

**DOI:** 10.3389/fncel.2021.688243

**Published:** 2021-10-21

**Authors:** Mingchao Li, Matthew C. Banton, Qing Min, David B. Parkinson, Xinpeng Dun

**Affiliations:** ^1^Department of Neurology, The Affiliated Huai’an No.1 People’s Hospital of Nanjing Medical University, Huai’an, China; ^2^School of Biomedical Science, Faculty of Health, University of Plymouth, Plymouth, United Kingdom; ^3^School of Pharmacy, Hubei University of Science and Technology, Xianning, China; ^4^Peninsula Medical School, Faculty of Health, University of Plymouth, Plymouth, United Kingdom; ^5^The Co-innovation Center of Neuroregeneration, Nantong University, Nantong, China

**Keywords:** peripheral nerve injury, transcription factors, upregulation, scRNA-seq, expression

## Abstract

Following peripheral nerve injury, transcription factors upregulated in the distal nerve play essential roles in Schwann cell reprogramming, fibroblast activation and immune cell function to create a permissive distal nerve environment for axonal regrowth. In this report, we first analysed four microarray data sets to identify transcription factors that have at least twofold upregulation in the mouse distal nerve stump at day 3 and day 7 post-injury. Next, we compared their relative mRNA levels through the analysis of an available bulk mRNA sequencing data set at day 5 post-injury. We then investigated the expression of identified TFs in analysed single-cell RNA sequencing data sets for the distal nerve at day 3 and day 9 post-injury. These analyses identified 55 transcription factors that have at least twofold upregulation in the distal nerve following mouse sciatic nerve injury. Expression profile for the identified 55 transcription factors in cells of the distal nerve stump was further analysed on the scRNA-seq data. Transcription factor network and functional analysis were performed in Schwann cells. We also validated the expression pattern of Jun, Junb, Runx1, Runx2, and Sox2 in the mouse distal nerve stump by immunostaining. The findings from our study not only could be used to understand the function of key transcription factors in peripheral nerve regeneration but also could be used to facilitate experimental design for future studies to investigate the function of individual TFs in peripheral nerve regeneration.

## Introduction

Transcription factors (TFs) are DNA binding proteins that drive various biological processes such as embryonic development, tissue identity maintenance and tissue repair following injury (Zhou et al., 2017; Lambert et al., 2018). The effects of TFs are amplified as one TF may bind to multiple promoter and enhancer regions and control the up- or downregulation of a large number of target genes (Zhou et al., 2017; Lambert et al., 2018). TFs constitute the second largest gene family in the mammalian genome, but so far only a few of them have been studied in relation to their role in peripheral nerve regeneration (Arthur-Farraj et al., 2012; Fontana et al., 2012; Patodia and Raivich, 2012; Quintes et al., 2016; Mindos et al., 2017; Roberts et al., 2017; Dun et al., 2019). Injury to a peripheral nerve results in a rapid Schwann cell reprogramming, mesenchymal cell activation and resident macrophage phenotype transition in the distal nerve stump (Jessen and Mirsky, 2016, 2019; Carr et al., 2019; Toma et al., 2020). Immune cells such as neutrophils, macrophages, mast cells, T cells, NK cells, and B cells are all recruited into the distal nerve stump follow injury (Martini et al., 2008), and recruited immune cells are reprogrammed into a repair immune cell phenotype by the distal nerve regenerative microenvironment (Ydens et al., 2012, 2020; Davies et al., 2019). The upregulation TFs in cells of the distal nerve play key roles in cell reprogramming, activation and phenotype transition to create a permissive distal nerve environment for axonal regrowth (Jessen and Mirsky, 2008; Arthur-Farraj et al., 2012; Doddrell et al., 2012; Fontana et al., 2012; Patodia and Raivich, 2012; Mindos et al., 2017; Roberts et al., 2017). One of the best-studied TFs in peripheral nerve regeneration is Jun and studies have shown that the upregulation of Jun in Schwann cells plays an essential role in reprograming adult Schwann cells into repair Schwann cells following peripheral nerve injury (Parkinson et al., 2008; Arthur-Farraj et al., 2012; Fontana et al., 2012).

Previous gene expression profiling studies using cDNA microarray and bulk mRNA sequencing technologies have identified hundreds of genes including TFs that are upregulated in the distal nerve following mouse sciatic nerve injury (Barrette et al., 2010; Arthur-Farraj et al., 2012; Clements et al., 2017; Pan et al., 2017; Norrmen et al., 2018). However, cDNA microarray and bulk mRNA sequencing studies are unable to map upregulated genes to different cell types in the distal nerve stump. Therefore, immunohistochemistry or *in situ* hybridisation techniques are often used to identify the cell types of an upregulated gene (Carr et al., 2017; Dun et al., 2019; Chen et al., 2020). Recent advances in single-cell RNA sequencing (scRNA-seq) technologies and the development of bioinformatic pipelines have enabled us to rapidly obtain cell type specific gene expression profiles in large numbers of individual cells (Chen et al., 2019). scRNA-seq technologies have been widely used in different research fields to reveal cell populations, to uncover regulatory relationships between genes, and to track the trajectories of distinct cell lineages (Hwang et al., 2018). However, this technology has only recently been applied to study cell types and gene expression profiles in the mouse distal nerve stump (Carr et al., 2019; Toma et al., 2020; Ydens et al., 2020). So far, only the expression profile of extracellular signalling molecules have been mapped to cells in the distal nerve stump by scRNA-seq data analysis (Toma et al., 2020).

One of our research interests is to study the function of TFs in peripheral nerve regeneration (Doddrell et al., 2012, 2013; Mindos et al., 2017; Roberts et al., 2017; Dun et al., 2019). In this report, we first analysed microarray and bulk mRNA sequencing data sets and identified 55 TFs with at least twofold upregulation in the mouse distal nerve stump at day 3 and day 7 post-injury. Next, we examined their expression profile in cells of injured mouse sciatic nerve using analysed scRNA-seq data sets for the mouse distal sciatic nerve at day 3 and day 9 post-injury (Carr et al., 2019; Toma et al., 2020). Following this analysis, we validated the expression pattern of Jun, Junb, Runx1, Runx2, and Sox2 in the mouse distal nerve stump by immunostaining, and performed transcription factor network and functional analysis in Schwann cells on the scRNA-seq data. The findings from our analysis could not only be used to understand cell specific functions of key transcription factors in peripheral nerve regeneration but also could be used to identify the function of individual TFs in peripheral nerve regeneration.

## Materials and Methods

### Data Mining of Transcription Factor Upregulation in the Mouse Distal Sciatic Nerve

Differentially expressed genes in the mouse distal sciatic nerve at day 3 and day 7 post-injury were obtained by analysing four microarray data sets from the NCBI gene expression omnibus (GEO) using NCBI online tool GEO2R. Affymetrix gene chip microarray data sets used in this study are GSE22291 (Barrette et al., 2010), GSE38693 (Arthur-Farraj et al., 2012), GSE74087 (Pan et al., 2017), and GSE146958 (Toma et al., 2020). Differentially upregulated genes were selected with a cut off value of LogFC ≥ 1. TFs were then manually searched within differentially expressed genes using a mammalian TF library reported by Lambert et al. (2018). Relative mRNA expression levels were analysed from the bulk mRNA sequencing data set GSE108231 (Norrmen et al., 2018).

### Computational Analysis of Single-Cell RNA Sequencing Datasets

scRNA-seq data GSE147285 for intact mouse sciatic nerve and day 3 post-injury distal nerve (Toma et al., 2020), and data set GSE120678 for day 9 post-injury distal nerve (Carr et al., 2019) were downloaded from the NCBI GEO database. Datasets were analysed using the Seurat v.3.2.1^1^ and sctransform v.0.3 R packages using R v.4.0.2. Quality control plots of number of features, counts and percentage mitochondrial content per cell were plotted for each data set and used to determine filtering conditions. For the quality control of intact mouse sciatic nerve data set, cells were filtered using the following conditions: number of features per cell 200–6000 and percent mitochondrial DNA content per cell <8%. For the quality control of the 3 days post-injury nerve data set, cells were filtered using the following conditions: number of features per cell 200–6000 and percent mitochondrial DNA content per cell <6%. For the quality control of 9 days post-injury nerve data set, cells were filtered using the following conditions: number of features per cell 200–4000 and percent mitochondrial DNA content per cell <8%.

Filtered cell data were normalised, variable genes identified and data scaled using SCTransform, a recently published highly effective method for removing technical artefacts from scRNAseq data while retaining biological heterogeneity (Hafemeister and Satija, 2019). The dimensionality of the dataset was determined using elbow plots to identify the appropriate number of principal components used for clustering. Cell clustering was performed using the FindNeighbors and FindCluster functions in Seurat. Differentially expressed genes were identified using the FindAllMarkers Seurat function using the Wilcoxon rank sum test for genes with a minimum 0.25 log fold change between clusters and expression in at least 10% of cells within the clusters. To annotate the clusters, genes differentially expressed in a one vs. all cluster comparison were queried for known expression in a literature search and gene expression plotted. Cell clustering was visualised using t-distributed stochastic neighbour embedding (tSNE) using the FeaturePlot function in Seurat. Dot plots for TFs were also plotted using Seurat specific functions. Gene expression abundance was calculated by Seurat to show the percentage of cell expression in a cluster and the average expression value of a gene in the same cell cluster. Transcription factor network analysis was performed on the scRNA-seq data using an R package SCENIC. Gene enrichment analysis was performed using Ingenuity.

### Marker Genes for the Identification of Cell Clusters

Cell clusters were identified based on the use of the following established marker genes for cell types of mouse sciatic nerves (Evrard et al., 2018; Zhao et al., 2018; Carr et al., 2019; Renthal et al., 2020; Toma et al., 2020; Wolbert et al., 2020; Xie et al., 2020; Ydens et al., 2020). Egfl7, Ecscr, Pecam1/Cd31, Tie1, Emcn, Cdh5, and Esam for endothelial cells. Lyve1, Mmrn1, Flt4, and Prox1 for lymphatic endothelial cells. Des, Tpm2, Myh11, Acta2, Mylk, Myom1, and Myocd for vascular smooth muscle (VSM) cells. Rgs5, Kcnj8, and Pdgfrb for pericytes. Sox10, Plp1, and S100b for Schwann cells. Mbp, Mpz, Mag, and Egr2 for myelinating Schwann cells. Dcn, Mfap5, Serpinf1, and Gsn for fibroblasts. Sfrp2, Dpt, Pcolce2, Adamts5, Pi16, Sfrp4, Prrx1, Comp, and Ly6c1 for epineurial fibroblasts. Cldn1/claudin-1, Lypd2, Ntn4, Msln, Ntng1, Slc2a1/Glut1, and Mpzl2 for perineurial cells. Sox9, Osr2, Wif1, Abca9, Cdkn2a, Cdkn2b, and Plxdc1 for endoneurial fibroblasts. Dlk1, Mest, Cilp, Tnc, Plagl1, and Ptn for differentiating fibroblasts. Ptprc/CD45 and Cd52 as general markers for immune cells. Aif1/Iba1, Cd68, Mrc1/Cd206, and Adgre1/F4/80 for macrophages. S100a8, S100a9, Cxcr2, and Cxcl2 for neutrophils. Cma1, Mcpt4, Mcpt1, and Kit for mast cells. Cd3g, Cxcr6, Trac, and Cd3e for T cells. Nkg7, Klrk1, and Ncr1 for natural killer (NK) cells. Bank1, Cbfa2t3, Taok3, Ms4a1, Cd19, and Cd79a for B cells (Evrard et al., 2018; Zhao et al., 2018; Carr et al., 2019; Renthal et al., 2020; Toma et al., 2020; Wolbert et al., 2020; Xie et al., 2020; Ydens et al., 2020).

### Peripheral Nerve Surgery

Six two-month old PLP-GFP mice were used in the study, Schwann cells express GFP in PLP-GFP mice (Mallon et al., 2002; Dun et al., 2019). All work involving animals was carried out according to Home Office regulations under the United Kingdom Animals (Scientific Procedures) Act 1986. Ethical approval for all experiments was granted by the Plymouth University Animal Welfare and Ethical Review Board. Mice were housed in a controlled laboratory environment (temperature 22 ± 2°C, humidity 50–60%, 12−h light/dark cycle), and fed with standard rodent diet and water *ad libitum*. For sciatic nerve transection injury, six male mice were anaesthetised with isoflurane, the right sciatic nerve was exposed and transected at approximately 0.5 cm proximal to the nerve trifurcation site and no re-anastamosis of the severed nerve was performed. Overlying muscle was sutured and the skin was closed with an Autoclip applier. Mice undergoing surgery were given appropriate post-operative analgesia, an 0.05% bupivacaine solution, topically applied above the muscle suture before applying surgical clips. Meloxicam injections (5 mg/kg) were given just before recovery from anaesthetic. Mice undergoing surgery were given nesting material and cage enrichment to minimise risk of autotomy. Animals under surgery were monitored daily. At day 3 and day 9 post-surgery, animals were euthanased humanely using carbon dioxide in accordance with United Kingdom Home Office regulations and nerve tissue removed for analysis.

### Immunohistochemistry

Sciatic nerves were dissected out and fixed for 5 h in 4% paraformaldehyde (in PBS, PH7.2) at 4°C. Nerves were then washed in PBS (3 × 10 min) and dehydrated in 30% sucrose (in PBS) overnight at 4°C. Subsequently, nerves were embedded in OCT medium, frozen, and sectioned on a cryostat at a thickness of 12 μm. Sections were permeabilised with 0.25% Triton X-100 plus 1% bovine serum albumin (BSA) in PBS for 45 min and then blocked with blocking buffer (3% BSA plus 0.05% Triton X-100 in PBS) for 1 h at room temperature. Sections were incubated with Jun (Cell Signalling, #9165S), Junb (Cell Signalling, #3753S), Sox2 (Cell Signalling, #2748), Runx2 (Cell Signalling, #8486), or Runx1 (Cell Signalling, #4334) primary antibody (1:100 diluted in blocking buffer) overnight at 4°C. The next day, sections were washed with PBS (3 × 10 min) and then incubated with donkey anti-rabbit secondary antibody conjugated with Alexa Fluor 568 (1:300 diluted in blocking buffer) 1 h at room temperature. Hoechst dye (1:500) was also added into secondary antibody solution in order to label cell nuclei. Finally, sections were washed with PBS (3 × 10 min) and mounted with Citifluor (Agar Scientific, R1320) for imaging with a LeicaSPE confocal microscope.

### Statistical Analysis

*p* values for genes of microarray data set analysis were obtained following online GEO2R tool analysis. Statistical significance for data set GSE108231 was analysed using the Student’s *t*-test by comparing the control group with the 5 day crush group (*n* = 3). The value of mRNA counts (normalised count) are represented in the Figure as mean value ± SEM.

## Results

### Identification of Transcription Factors With at Least Twofold Upregulation in the Mouse Distal Nerve Stump at Day 3 and Day 7 Post-injury

Affymetrix gene chip microarray data sets GSE22291 (Barrette et al., 2010), GSE38693 (Arthur-Farraj et al., 2012), GSE74087 (Pan et al., 2017), and GSE146958 (Toma et al., 2020) were analysed using the NCBI online tool GEO2R to obtain differentially expressed genes at day 3 and day 7 post-injury. LogFC ≥ 1 was used as the cut off value to select genes with at least a twofold upregulation. TFs with at least a twofold upregulation were then manually searched and identified using a mammalian TFs library (1639 TFs) reported by Lambert et al. (2018). We analysed three microarray data sets for each time point, a TF showing upregulation at least in two data sets was selected for further analysis (Supplementary Tables 1, 2). This analysis identified 31 TFs that are upregulated in the mouse distal nerve stump at both day 3 and day 7 post-injury, 10 TFs that are upregulated only at day 3 post-injury and 15 TFs that are upregulated only at day 7 post-injury (Table 1). In total, 56 TFs were identified with at least a twofold upregulation in the distal nerve following mouse sciatic nerve injury.

**TABLE 1:** TFs with at least twofold upregulation post-injury in the mouse distal sciatic nerve.

Time points post-injury	Gene name (logFC ≥ 1)
3 and 7 days (31 TFs)	Aebp1, Atf3, Cenpa, Cenpt, Dnmt1, E2f1, E2f3, E2f7, E2f8, Egr3, Ets1, Fos, Fosl1, Foxm1, Hhex, Hivep3, Hmga1, Hmga2, Ikzf1, Irf8, Jun, Mafb, Mxd3, Nr1h4, Runx1, Runx2, Sox2, Sox4, Tcfl5, Tfec, Tgif1
3 days only (10 TFs)	Cebpb, Foxq1, Irf5, Irf7, Junb, Myc, Nfe2, Spi1, Thyn1, Zbed4
7 days only (15 TFs)	Bach1, Bach2, Crem, Fli1, Fosl2, Glis3, Klf10, Myod1, Nfya, Olig1, Plagl1, Tead2, Tfap2a, Trps1, Zeb1

### Expression Levels of Identified Transcription Factors in the Distal Nerve

**FIGURE 1:**
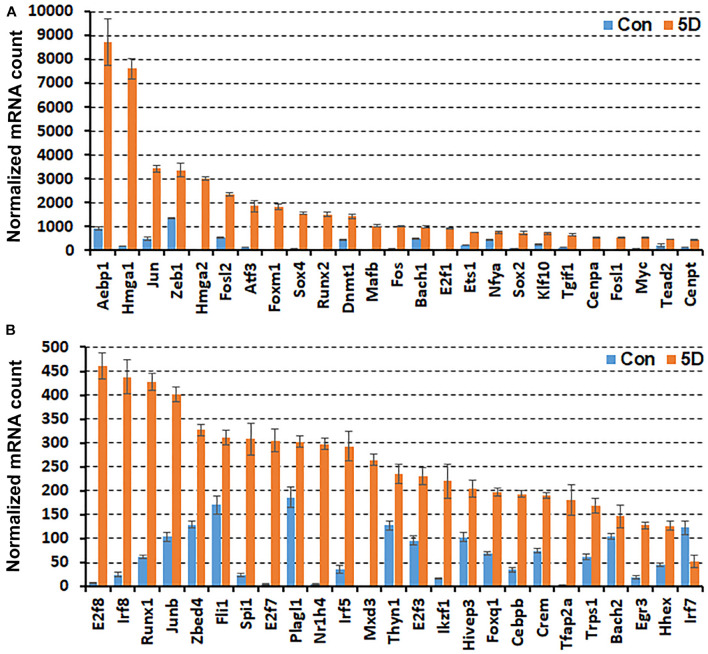
Comparison of normalised mRNA counts for above identified TFs in mouse distal nerve following sciatic nerve crush injury by the analysis of bulk mRNA sequencing data set GSE108231. TFs are arranged according to their normalised mRNA count from highest to lowest in the distal nerve at day 5 post-injury. **(A)** mRNA counts for TFs bigger than 460 at day 5 post-injury. **(B)** mRNA counts for TFs smaller than 460 at day 5 post-injury. Con (in blue): control contralateral nerve. 5D (in yellow): distal nerve at 5 day post-crush injury. Glis3, Myod1, Nfe2, Olig1, Tcfl5, and Tfec mRNAs were all undetectable in this data set (*n* = 3, all *p* values < 0.05).

While scRNA-seq data for injured and intact peripheral nerves is available, it takes several hours to obtain single cell suspensions from peripheral nerves for subsequent scRNA-seq analysis (Carr et al., 2019; Toma et al., 2020; Wolbert et al., 2020). The *in vitro* tissue enzyme digestion and single cell separation procedures could alter the levels of *in vivo* gene expression (Vanlandewijck et al., 2018; Xu et al., 2019). We therefore analysed the bulk RNA-seq data set GSE108231 to compare the relative mRNA levels for the above identified 56 TFs in injured mouse sciatic nerve. The GSE108231 data set performed bulk mRNA sequencing to analyse gene expression in intact mouse sciatic nerve and in the distal nerve at day 5 following sciatic nerve crush injury (Norrmen et al., 2018). Analysing the bulk mRNA sequencing data set revealed that mRNA of 50 of the identified 56 TFs above are detectable in the distal nerve, but Glis3, Myod1, Nfe2, Olig1, Tcfl5, and Tfec mRNA are undetectable in this data set for both intact nerves and the distal nerve stump at day 5 post-injury (Figure 1). All of these 50 TFs (except Irf7) show upregulation in the distal nerve stump at day 5 post-injury compared to their mRNA levels in intact nerve (Figure 1). The above microarray data analysis showed that Irf7 was only upregulated at day 3 post-injury. Therefore, Irf7 mRNA levels may decrease rapidly between 3 and 5 days post-injury. This analysis also indicates that Glis3, Myod1, Nfe2, Olig1, Tcfl5, and Tfec are either not expressed in the distal nerve stump at day 5 post-injury or they have a very low level of expression that could be excluded during data normalisation.

In the distal nerve at day 5 post-injury, Aebp1, Hmga1, Jun, Zeb1, Hmga2, and Fosl2 have very high levels of expression and their normalised mRNA counts are greater than 2000 (Figure 1A). Atf3, Foxm1, Sox4, Runx2, Dnmt1, Mafb, and Fos show the next highest levels of expression with their normalised mRNA counts between 1000 and 2000 (Figure 1A). The normalised mRNA counts for Bach1, E2f1, Ets1, Nfya, Sox2, Klf10, Tgif1, Cenpa, Fosl1, Myc, Tead2, Cenpt, E2f8, Irf8, Runx1, Junb, Zbed4, Fli1, Spi1, E2f7, Plagl1, Nr1h4, Irf5, Mxd3, Thyn1, E2f3, Ikzf1, and Hivep3 range between 200 and 1000 (Figures 1A,B). However, the normalised mRNA counts for Foxq1, Cebpb, Crem, Tfap2a, Trps1, Bach2, Egr3, Hhex, and Irf7 are all below 200 despite their significant upregulation (Figure 1B), indicating that their mRNA levels are very low within injured nerve at day 5 post-injury.

### Expression of Identified Transcription Factors in Different Cell Types of Mouse Distal Sciatic Nerve Stump

**FIGURE 2:**
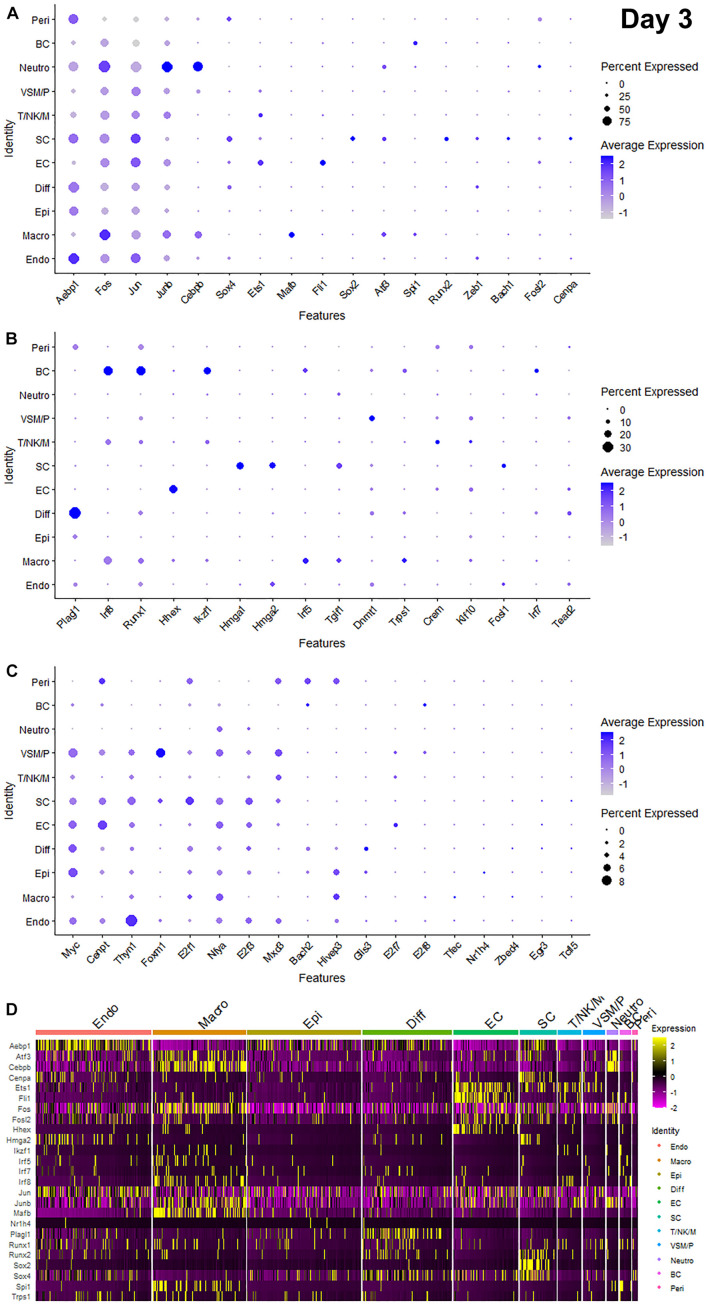
Dotplots and heatmap showing 51 TFs that are up-regulated in the distal nerve at day 3 post-injury. **(A)** Dotplots for Aebp1, Fos, Jun, Junb, Cebpb, Sox4, Ets1, Mafb, Fli1, Sox2, Atf3, Spi1, Runx2, Zeb1, Bach1, Fosl2, and Cenpa which have more than 25% expression at least in one cell cluster. **(B)** Dotplots for Plagl1, Irf8, Runx1, Hhex, Ikzf1, Hmga1, Hmga2, Irf5, Tgif1, Dnmt1, Trps1, Crem, Klf10, Fosl1, Irf7, and Tead2 which have expression between 8 and 25% at least in one cell cluster. **(C)** Dotplots for Myc, Cenpt, Thyn1, Foxm1, E2f1, Nfya, E2f3, Mxd3, Bach2, Hivep3, Glis3, E2f7, E2f8, Tfec, Nr1h4, Zbed4, Egr3, and Tcfl5 which have expression below 8% in any cell cluster. **(D)** Heatmap for Aebp1, Atf3, Cebpb, Cenpa, Ets1, Fli1, Fos, Fosl2, Hhex, Hmga2, Ikzf1, Irf5, Irf7, Irf8, Jun, Junb, Mafb, Nr1h4, Plagl1, Runx1, Runx2, Sox2, Sox4, Spi1, and Trps1. Diff: Differentiating fibroblasts; Endo: Endoneurial fibroblasts; Peri: Perineurial fibroblasts; Epi: Epineurial fibroblasts; SC: Schwann cells; EC: Endothelial cells; VSM/P: Vascular smooth muscle cells and pericytes; Neutro: Neutrophils; Macro: Macrophages; BC: B cells; T/NK/M: T cells, NK cells, and mast cells.

**FIGURE 3:**
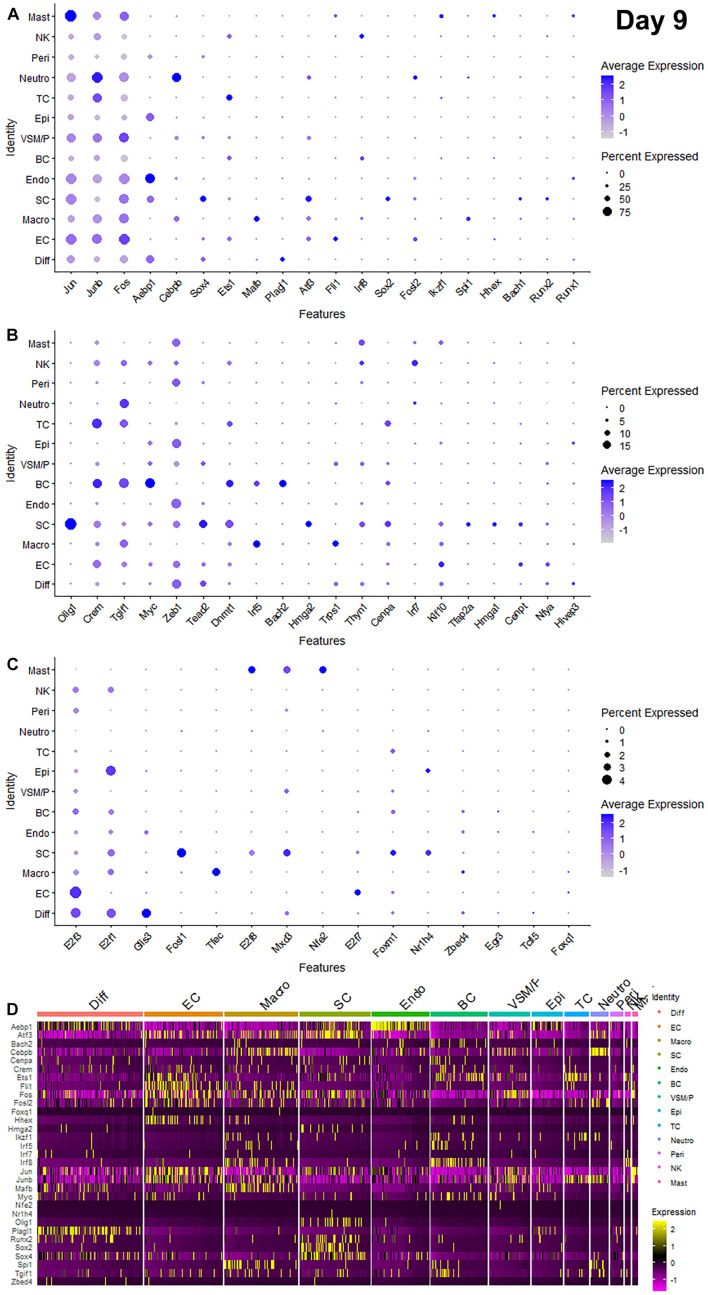
Dotplots and heatmap showing 55 TFs that are up-regulated in the distal nerve at day 9 post-injury. **(A)** Dotplots for Jun, Junb, Fos, Aebp1, Cebpb, Sox4, Ets1, Mafb, Plagl1, Atf3, Fli1, Irf8, Sox2, Fosl2, Ikzf1, Spi1, Hhex, Bach1, Runx2, and Runx1 which have more than 20% expression at least in one cell cluster. **(B)** Dotplots for Olig1, Crem, Tgif1, Myc, Zeb1, Tead2, Dnmt1, Irf5, Bach2, Hmga2, Trps1, Thyn1, Cenpa, Irf7, Klf10, Tfap2a, Hmga1, Cenpt, Nfya, and Hivep3 which have expression between 5 and 20% at least in one cell cluster. **(C)** Dotplots for E2f3, E2f1, Glis3, Fosl1, Tfec, E2f8, Mxd3, Nfe2, E2f7, Foxm1, Nr1h4, Zbed4, Egr3, Tcfl5, and Foxq1 which have expression below 5% in any cell cluster. **(D)** Heatmap for Aebp1, Atf3, Bach2, Cebpb, Cenpa, Crem, Ets1, Fli1, Fos, Fosl2, Foxq1, Hhex, Hmga2, Ikzf1, Irf5, Irf7, Irf8, Jun, Junb, Mafb, Myc, Nfe2, Nr1h4, Olig1, Plagl1, Runx2, Sox2, Sox4, Spi1, Tgif1, and Zbed4. Diff: Differentiating fibroblasts; Endo: Endoneurial fibroblasts; Peri: Perineurial fibroblasts; Epi: Epineurial fibroblasts; SC: Schwann cells; EC: Endothelial cells; VSM/P: Vascular smooth muscle cells and pericytes; Neutro: Neutrophils; Macro: Macrophages; BC: B cells; TC: T cells; NK: NK cells; and Mast: mast cells.

Recently, we reported our analysis of publicly available scRNA-seq data for injured mouse sciatic nerve at day 3 and day 9 post-injury (Carr et al., 2019; Toma et al., 2020; Chen et al., 2021). We then examined the expression of above 56 TFs in our analysed scRNA-seq data. This analysis revealed that 51 of the 56 TFs are expressed in the scRNA-seq data GSE147285 at day 3 post-injury (Figure 2), Foxq1, Myod1, Nfe2, Olig1, and Tfap2a were undetectable in this data set. 55 TFs are expressed in the scRNA-seq data GSE120678 at day 9 post-injury (Figure 3), only Myod1 was undetectable in this data set. Next, we investigated their expression in cells of mouse distal sciatic nerve stump using analysed scRNA-seq data sets at both day 3 and day 9 post-injury. Among all the identified TFs, Aebp1, Fos, Jun, and Junb are upregulated in most cell types of the distal nerve stump at both day 3 and day 9 post-injury (Figures 2, 3). Aebp1 shows strong up-regulation in fibroblast and Schwann cells. Fos shows strong up-regulation in immune cells and cells associated with blood vessels. Jun shows high up-regulation in Schwann cells and mast cells, and Junb is highly expressed in neutrophils (Figures 2, 3).

Schwann cells are the most abundant cell type in the peripheral nerves, and TFs play a key role in re-programming adult Schwann cells into repair Schwann cells to promote peripheral nerve regeneration (Jessen and Mirsky, 2016). Our examination revealed that Bach1, Fosl1, Hmga1, Hmga2, Sox4, and Runx2 are all highly expressed in Schwann cells at both day 3 and day 9 post-injury (Figures 2, 3). Sox2 shows exclusively Schwann cell expression at day 3 and day 9 post-injury (Figures 2, 3). Olig1 and Tfap2a are not expressed in nerves at day 3 post-injury but both are solely upregulated in Schwann cells at day 9 post-injury (Figure 3). The microarray data set analysis also showed that Olig1 and Tfap2a are only upregulated in the distal nerve at day 7 post-injury. Our analysis also showed that Cepna, E2f1, E2f3, Jun, Tcfl5, and Tgif1 have their highest expression in Schwann cells at day 3 post-injury comparing to other cell types in the distal nerve stump (Figure 2). At day 9 post-injury, Atf3, Bach1, Cepna, Foxm1, Mdx3, and Tead2 have their highest expression in Schwann cells comparing to other cell types in the distal nerve stump (Figure 3).

Fibroblasts are the second most abundant cell population in the peripheral nerves (Carr et al., 2019; Toma et al., 2020; Wolbert et al., 2020). According to their anatomical location and gene expression profile in the injured nerve, they have been sub-grouped into endoneurial, perineurial, epineurial, and differentiating fibroblasts (Carr et al., 2019; Toma et al., 2020; Chen et al., 2021). Among all the TFs that expressed in fibroblasts, only Glis3 and Plagl1 show fibroblast specific expression at both day 3 and day 9 post-injury. More fibroblasts express Plagl1 than Glis3 at both day 3 and day 9 post-injury (Figures 2, 3). Glis3 and Plagl1 have their highest expression in differentiating fibroblasts at both at day 3 and day 9 post-injury comparing to other cell types in the distal nerve stump (Figures 2, 3). Our analysis shows that Zeb1 at day 3 post-injury (Figure 2), Hivep3 and Tcfl5 at day 9 post-injury (Figure 3) have their highest expression in differentiating fibroblasts. Although it is not at their highest level of expression, Aebp1, Fos, Jun, Junb, and Sox4 at both day 3 and day 9 post-injury are highly expressed in differentiating fibroblasts at both day 3 and day 9 post-injury (Figures 2, 3 and Supplementary Tables 3, 4).

Aebp1 has the highest level of expression in endoneurial fibroblasts at both day 3 and day 9 post-injury (Figures 2, 3). Thyn1 at day 3 following injury (Figure 2) and Zeb1 at day 9 (Figure 3) following injury also show their highest expression in endoneurial fibroblasts. Cebpb, Fos, Jun, and Junb at both day 3 and day 9 post-injury are highly expressed in endoneurial fibroblasts (Figures 2, 3 and Supplementary Tables 3, 4). Hivep3, Zbed4, Nr1h4, and Myc show their highest expression in epineurial fibroblasts at day 3 post-injury (Figure 2). E2f1 shows its highest expression in epineurial fibroblasts at day 9 following injury (Figure 3). Aebp1, Fos, Jun, and Junb at both day 3 and day 9 post-injury are highly expressed in epineurial fibroblasts (Figures 2, 3 and Supplementary Tables 3, 4). Zeb1 is highly expressed in the injured nerves (Figure 1) and it is the only TF that shows high expression in perineurial fibroblasts (Figures 2, 3).

Cells of the blood supply system in the peripheral nerves include endothelial cells, VSM and pericytes (Stierli et al., 2018; Carr et al., 2019; Toma et al., 2020; Wolbert et al., 2020). Cenpt, E2f7, Fli1, Klf10, and Nfya have their highest expression in endothelial cells at both day 3 and day 9 post-injury comparing to other cell types in the distal nerve stump (Figures 2, 3). Egr3, Ets1, Hhex, and Tead2 show their highest expression in endothelial cells at day 3 post-injury (Figure 2), and E2f3, Fos, and Foxq1 show their highest expression in endothelial cells at day 9 post-injury (Figure 3). Despite not being at their highest level of expression in endothelial cells, Aebp1, Fos, Fosl2, Jun, Junb, and Sox4 at day 3 post-injury (Figure 2 and Supplementary Table 3) and Atf3, Ets1, Fil1, Fosl2, Jun, Junb, and Sox4 at day 9 post-injury (Figure 3 and Supplementary Table 4) are all highly expressed in endothelial cells. Dnmt1, Foxm1, and Mxd3, have their highest expression in VSM/pericytes at day 3 post-injury (Figure 2).

Immune cells that are recruited into the distal nerve stump following injury include neutrophils, macrophages, mast cells, T cells, NK cells, and B cells (Carr et al., 2019; Toma et al., 2020). Neutrophils are the earliest immune cells to be recruited into the injury site and the distal nerve stump (Lindborg et al., 2017). Cebpb, Fosl2, and Junb have their highest expression in neutrophils at both day 3 and day 9 post-injury (Figures 2, 3). Fos at day 3 post-injury also shows its highest expression in neutrophils (Figure 2). Macrophages including both resident macrophages and infiltrated macrophages are the largest immune cell population in the distal nerve stump (Zigmond and Echevarria, 2019). Mafb shows high macrophage specific expression at both day 3 and day 9 post-injury compared to other TFs that are expressed in macrophages (Figures 2, 3). Irf5, Tfec, and Trps1 also show high expression in macrophages at both day 3 and day 9 post-injury (Figures 2, 3). Atf3 at day 3 (Figure 2), Spil and Zebd4 at day 9 (Figure 3) post-injury also show their high expression in macrophages. Bach2, E2f8, Ikzf1, Irf7, Irf8, Runx1, and Spi1 have the highest expression in B cells at day 3 post-injury (Figure 2). In contrast, Bach2, Dnmt1, Egr3, Myc, and Tgif1 have highest expression in B cells at day 9 post-injury (Figure 3).

T cells, NK cells, and mast cells are not separated from each other in nerves at day 3 post-injury due to either their similar gene expression profile or low cell numbers in day 3 injured nerves (Chen et al., 2021). At day 3 post-injury, Crem shows high expression in the cluster of T, NK, and mast cells (Figure 2). T cells, NK cells, and mast cells were placed into three individual cell clusters at day 9 post-injury (Chen et al., 2021), Crem shows its high expression in T cells at day 9 post-injury (Figure 3). E2f8, Hhex, Ikzf1, Jun, Mxd3, Nfe2, Runx1, and Thyn1 all show their highest expression in mast cells at day 9 post-injury (Figure 3). Irf7 and Irf8 have their high expression in NK cells at day 9 post-injury (Figure 3). Cepna, Crem, and Ets1 have their highest expression in T cells at day 9 post-injury (Figure 3). At day 9 post-injury, Fil1, Fos, and Junb are also expressed in mast cells, Fos, Jun, Junb, and Tgif1 are also expressed in T cells and Ets1, Fos, Jun, and Junb are all highly expressed in NK cells (Figure 3 and Supplementary Table 4).

We summarise the cell types that show the highest level of expression at day 3 post-injury and day 9 post-injury for the identified 55 TFs in Table 2. We also provide Supplementary Tables to summarise their expression abundance in each cell type at day 3 (Supplementary Table 3) and day 9 post-injury (Supplementary Table 4).

**TABLE 2:** Cell types showing the highest level of expression.

TFs	Day 3	Day 9
Aebp1	Endoneurial fibroblasts	Endoneurial fibroblasts
Atf3	Macrophages	Endothelial cells
Bach1	Schwann cells	Schwann cells
Bach2	Perineurial fibroblasts	B cells
Cebpb	Neutrophils	Neutrophils
Cenpa	Schwann cells	T cells
Cenpt	Endothelial cells	Endothelial cells
Crem	Perineurial fibroblasts	T cells
Dnmt1	VSM, pericytes	Schwann cells
E2f1	Schwann cells	Epineurial fibroblasts
E2f3	Schwann cells	Endothelial cells
E2f7	Endothelial cells	Endothelial cells
E2f8	B cells	Mast cells
Egr3	Endothelial cells	B cells
Ets1	Endothelial cells	T cells
Fli1	Endothelial cells	Endothelial cells
Fos	Neutrophils	Endothelial cells
Fosl1	Schwann cells	Schwann cells
Fosl2	Neutrophils	Endothelial cells
Foxm1	VSM, pericytes	Schwann cells
Foxq1	No expression	Endothelial cells
Glis3	Differentiating fibroblasts	Differentiating fibroblasts
Hhex	Endothelial cells	Mast cells
Hivep3	Perineurial fibroblasts	Differentiating fibroblasts
Hmga1	Schwann cells	Schwann cells
Hmga2	Schwann cells	Schwann cells
Ikzf1	B cells	Mast cells
Irf5	Macrophages	Macrophages
Irf7	B cells	NK cells
Irf8	B cells	NK cells
Jun	Neutrophils	Mast cells
Junb	Neutrophils	Neutrophils
Klf10	Perineurial fibroblasts	Endothelial cells
Mafb	Macrophages	Macrophages
Mxd3	VSM, pericytes	Mast cells
Myc	Differentiating fibroblasts	B cells
Nfe2	No expression	Mast cells
Nfya	Endothelial cells	Endothelial cells
Nr1h4	Epineurial fibroblasts	Schwann cells
Olig1	No expression	Schwann cells
Plagl1	Differentiating fibroblasts	Differentiating fibroblasts
Runx1	B cells	Mast cells
Runx2	Schwann cells	Schwann cells
Sox2	Schwann cells	Schwann cells
Sox4	Schwann cells	Schwann cells
Spi1	B cells	Macrophages
Tead2	Differentiating fibroblasts	Schwann cells
Tcfl5	Schwann cells	Differentiating fibroblasts
Tfap2a	No expression	Schwann cells
Tfec	Macrophages	Macrophages
Thyn1	Endoneurial fibroblasts	Mast cells
Tgif1	Schwann cells	B cells
Trps1	Macrophages	Macrophages
Zbed4	Macrophages	Macrophages
Zeb1	Differentiating fibroblasts	Endoneurial fibroblasts

### Validation of Jun, Junb, Runx1, Runx2, and Sox2 Expression in Cells of the Distal Nerve Stump

**FIGURE 4:**
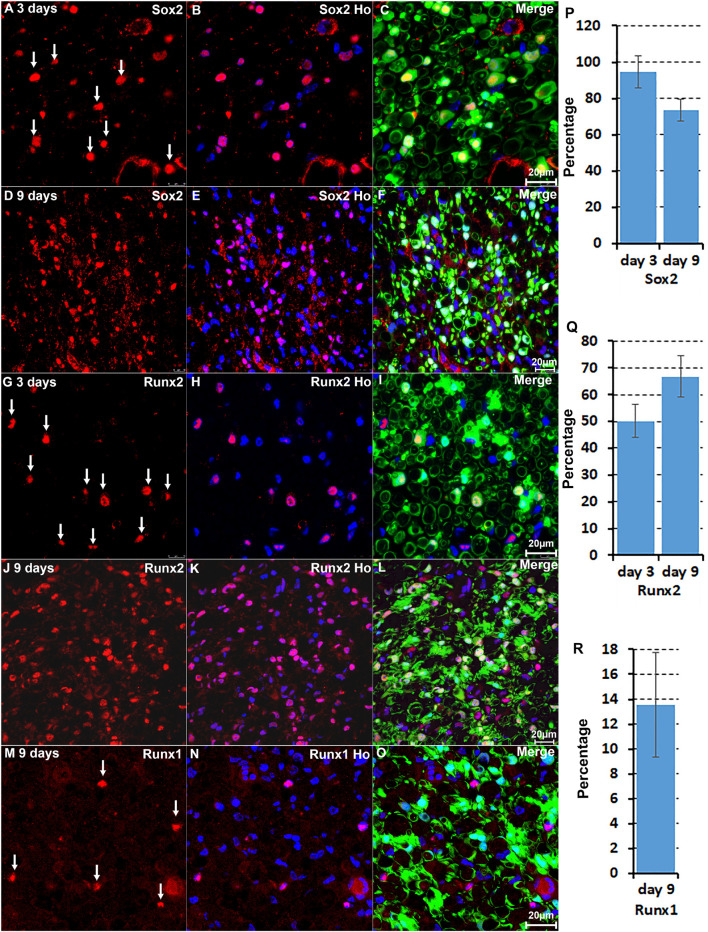
Immunostaining validates Sox2 and Runx2 expression in Schwann cells. **(A–C)** Sox2 expression in Schwann cells at day 3 post-injury. **(D–F)** Sox2 expression in Schwann cells at day 9 post-injury. **(G–I)** Runx2 expression in Schwann cells at day 3 post-injury. **(J–L)** Runx2 expression in Schwann cells at day 9 post-injury. **(M–O)** Runx1 expression in non-Schwann cells (GFP negative) at day 9 post-injury. **(P,Q)** Quantification of Runx2 and Sox2 expressing Schwann cells against the total number of Schwann cells in the distal nerve stump. **(R)** Quantification of Runx1 expressing cells against the total number of GFP negative cells in the distal nerve stump. For all panels, sections of distal nerve following injury were prepared from PLP-GFP transgenic mice that label Schwann cells with GFP. Scale bars in C, F, I, L, and O: 20 μm. Quantification data in **(P–R)** was shown as Mean ± SEM, *n* = 3.

We are interested in studying the function of TFs in Schwann cell biology (Parkinson et al., 2008; Doddrell et al., 2012, 2013; Mindos et al., 2017; Roberts et al., 2017; Dun et al., 2019). Our analysis revealed that the upregulation of Bach1, Fosl1, Hmga1, Hmga2, Sox2, and Runx2 are highly Schwann cell specific at both at day 3 and day 9 post-injury (Figures 2, 3). To validate this finding, we examined Runx2 and Sox2 expression on day 3 and day 9 post-injury mouse sciatic nerve sections from PLP-GFP mice, in which Schwann cells are GFP labelled (Mallon et al., 2002; Dun et al., 2019). The immunostaining results confirmed that both Runx2 and Sox2 are upregulated in Schwann cells at day 3 and day 9 following peripheral nerve injury (Figures 4A–L). Quantification of Runx2 and Sox2 expressing Schwann cells against the total number of Schwann cells in the distal nerve stump after immunostaining revealed that more than 90% Schwann cells express Sox2 at day 3 post-injury, and 73.3 ± 6.0% Schwann cells express Sox2 at day 9 post-injury (Figure 4P). About 50.1 ± 6.1% Schwann cells express Runx2 at day 3 post-injury, and 66.8 ± 7.7% Schwann cells express Runx2 at day 9 post-injury (Figure 4Q).

The microarray data analysis showed that Runx1, another Runx family transcription factor (Cohen, 2009; Otalora-Otalora et al., 2019), is also upregulated in the distal nerve stump (Table 1). In contrast to Runx2 expression in Schwann cells, Runx1 showed high expression in B cells at day 3 post-injury (Figure 2). At day 9 post-injury, Runx1 has the highest expression in mast cells (Figure 3). Because of the low number of mast cells in the nerve at day 3 post-injury (Carr et al., 2019; Toma et al., 2020; Chen et al., 2021), we then stained Runx1 on sciatic nerve sections from PLP-GFP mice at day 9 post-injury. The staining showed that Runx1 localizes in the cell nuclei but these cells are GFP negative cells (Figures 4M–O), confirming our finding from scRNA-seq data analysis that Runx1 is not expressed in Schwann cells. Quantification of Runx1 expressing cells against the total number of GFP negative cells in the distal nerve stump showed that 13.6 ± 4.2% none Schwann cells express Runx1 at day 9 post-injury (Figure 4R).

**FIGURE 5:**
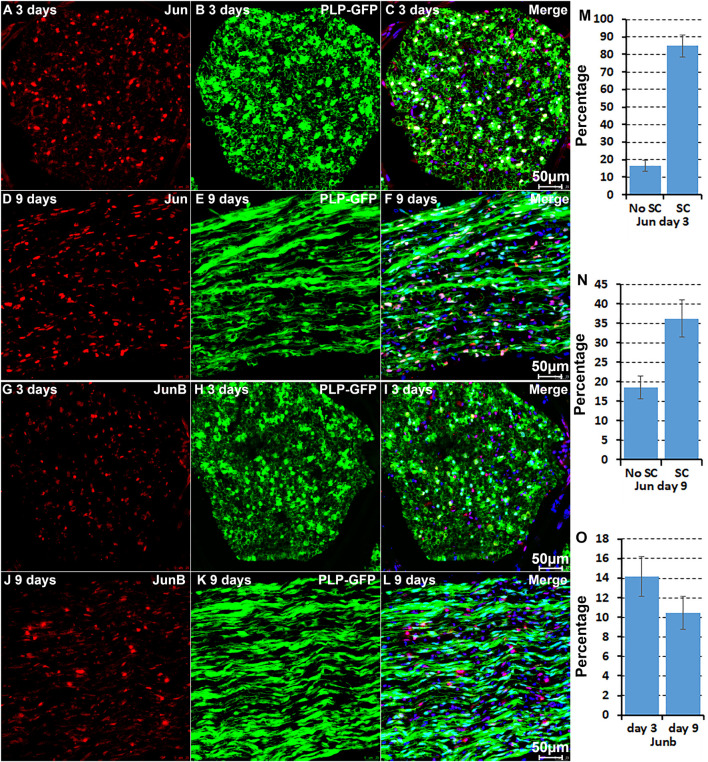
Immunostaining validates Jun and Junb expression in cells of the distal nerve stump. **(A–C)** Jun is primarily expressed in Schwann cells of the distal nerve at day 3 post-injury. **(D–F)** Jun expression in cells of the distal nerve at day 9 post-injury. **(G–I)** Junb expression in GFP negative cells of the distal nerve at day 3 post-injury. **(J–L)** Junb expression in GFP negative cells of the distal nerve at day 9 post-injury. **(M,N)** Quantification of Jun expressing in Schwann cells against the total number of Schwann cells in the distal nerve stump at day 3 and day 9 post-injury. **(O)** Quantification of Junb expressing cells against the total number of GFP negative cells in the distal nerve stump. For all panels, sections of distal nerve following injury were prepared from PLP-GFP transgenic mice that label Schwann cells with GFP. Scale bars in C, F, I, and L: 50 μm. Quantification data in **(M–O)** was shown as Mean ± SEM, *n* = 3.

We further performed immunostaining for Jun and Junb on sciatic nerve sections from PLP-GFP mice at day 3 and day 9 post-injury (Figure 5). Previous studies have shown a critical function of Jun in Schwann cells to promote peripheral nerve regeneration (Arthur-Farraj et al., 2012; Fontana et al., 2012). Our analysis indicated that Jun has the highest expression level in Schwann cells of the distal nerve at day 3 post-injury (Figure 2). Staining for Jun on the sciatic nerve sections from PLP-GFP mice at day 3 post-injury revealed that Jun is largely expressed in GFP positive Schwan cells at day 3 post-injury. Quantification data at day 3 post-injury showed that 84.8 ± 6.2% Schwan cells express Jun and only 16.4 ± 3.1% none Schwan cells express Jun (Figures 5A–C,M). At day 9 post-injury, our analysis showed that Jun retains its expression in Schwann cells but it is also highly expressed in B cells (Figure 3). The staining results for Jun also showed that 36.2 ± 4.7% Schwann cells continue to express Jun at day 9 post-injury, and 18.9 ± 2.9% none Schwann cells (GFP negative) express Jun (Figures 5D–F,N). Our analysis indicated that Junb is mainly expressed in immune cells of the distal nerve at both day 3 and day 9 post-injury (Figures 2, 3), and the staining results for Junb confirmed that Junb is expressed in GFP negative cells of the distal nerve. 14.2 ± 2.1% GFP negative cells at day 3 post-injury and 10.5 ± 1.7% GFP negative cells at day 9 post-injury express Junb (Figures 5G–L,O). Thus, the staining results for Jun, Junb, Runx1, Runx2, and Sox2 are all closely aligned to their expression pattern revealed by the scRNA-seq data analysis.

### Transcription Factor Network and Functional Analysis in Schwann Cells

**FIGURE 6:**
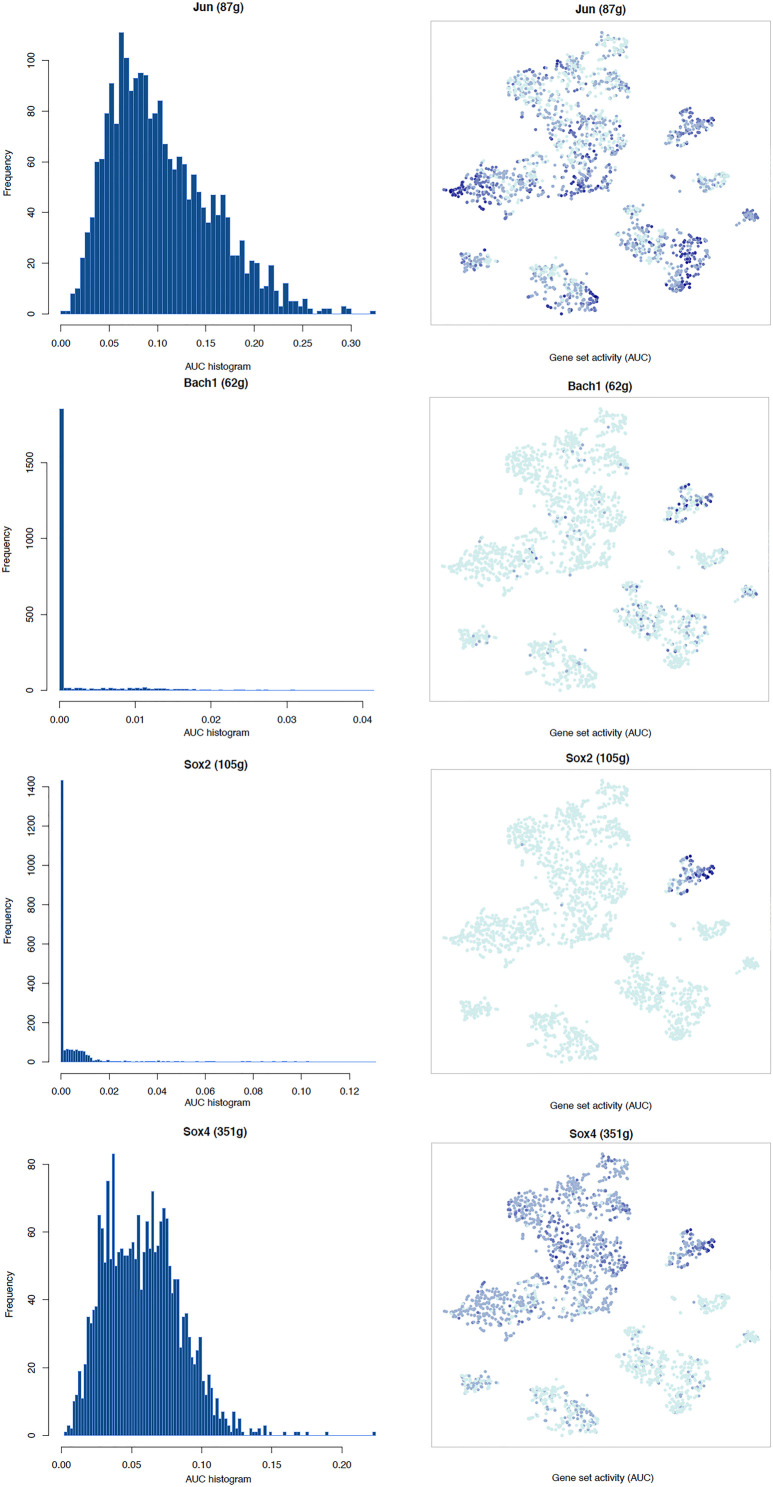
tSNE plots overlayed with regulon activity and histograms showing the distribution of regulon activity of Jun, Bach1, Sox2, and Sox4 in day 3 scRNA-seq data set.

**FIGURE 7:**
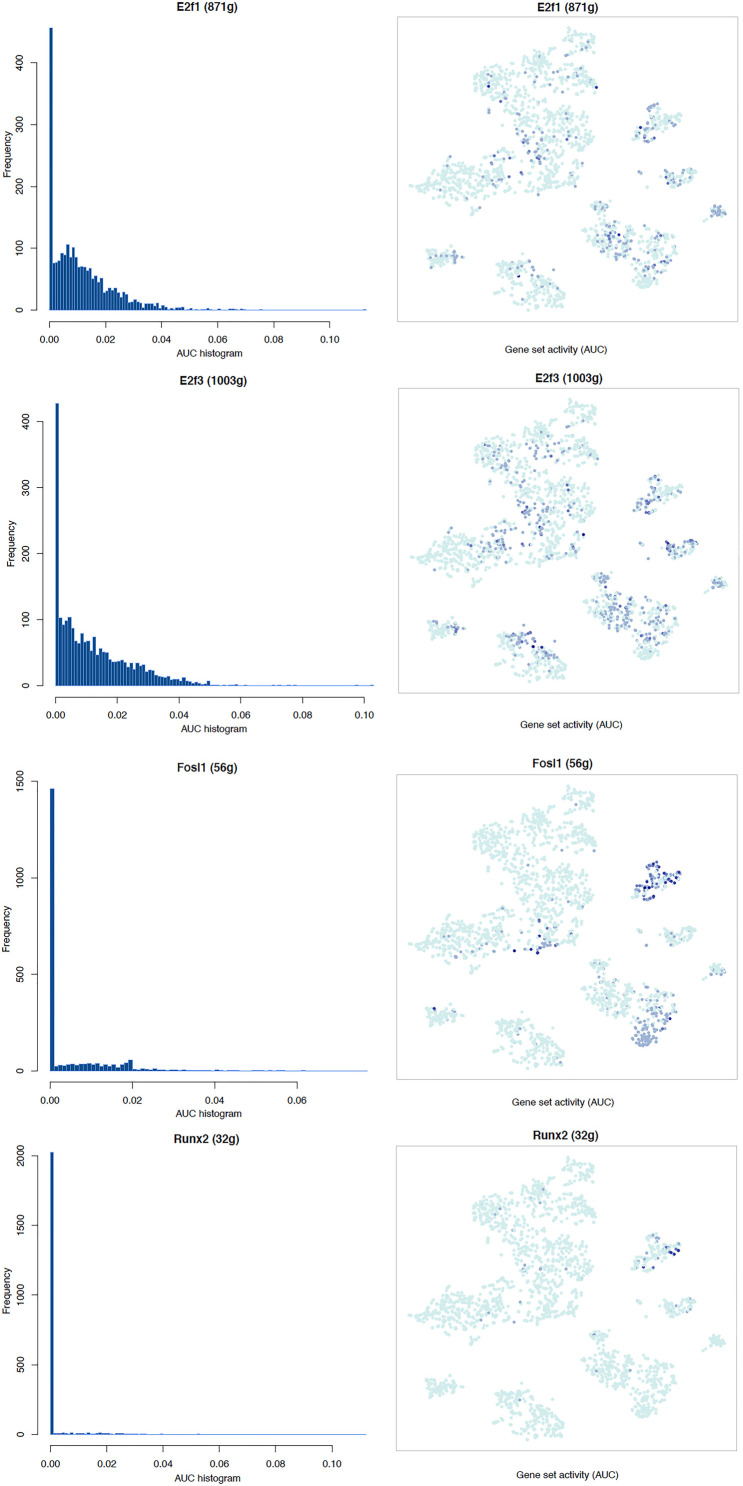
tSNE plots overlayed with regulon activity and histograms showing the distribution of regulon activity of E2f1, E2f3, Fosl1, and Runx2 in day 3 scRNA-seq data set.

**FIGURE 8:**
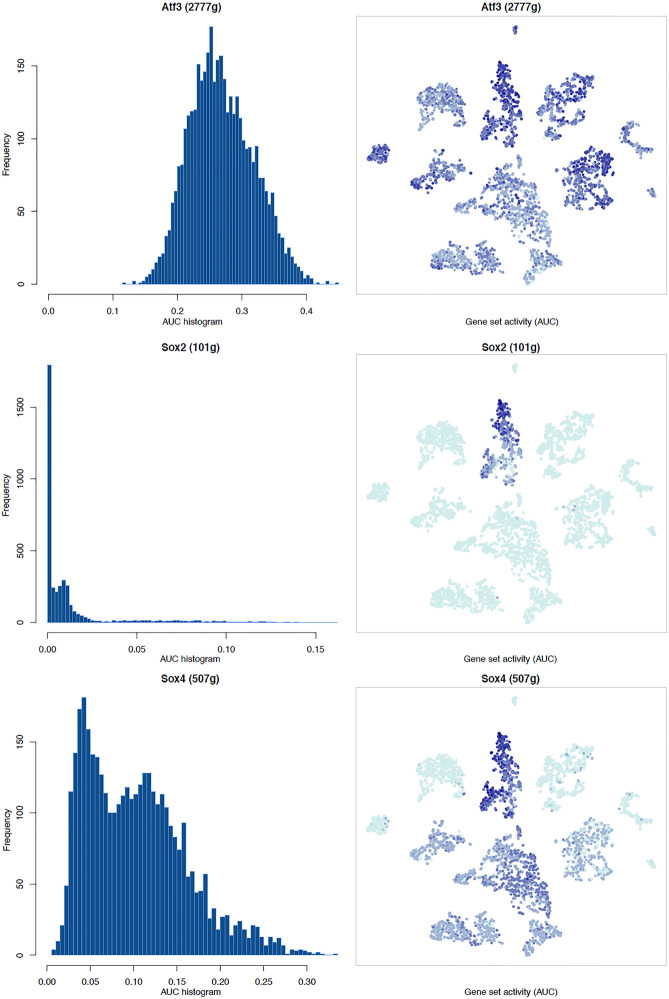
tSNE plots overlayed with regulon activity and histograms showing the distribution of regulon activity of Atf3, Sox2, and Sox4 in day 9 scRNA-seq data set.

**FIGURE 9:**
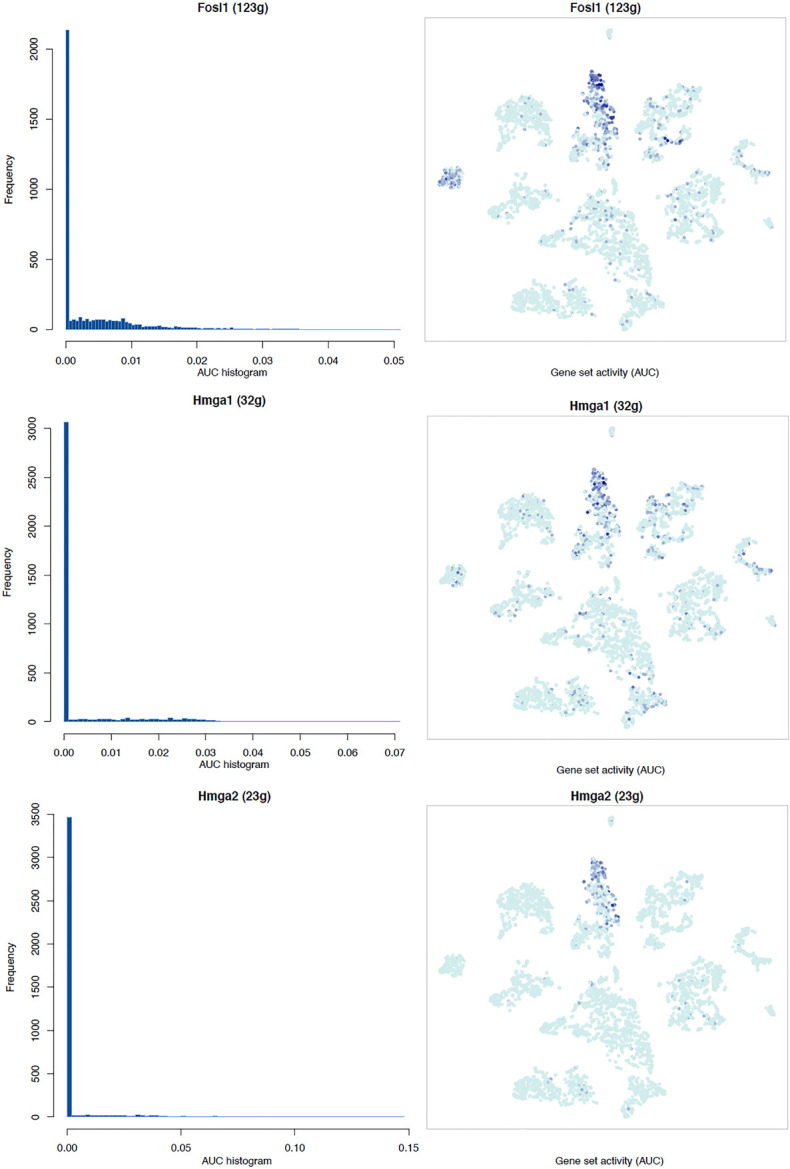
tSNE plots overlayed with regulon activity and histograms showing the distribution of regulon activity of Fosl1, Hmga1, and Hmga2 in day 9 scRNA-seq data set.

**FIGURE 10:**
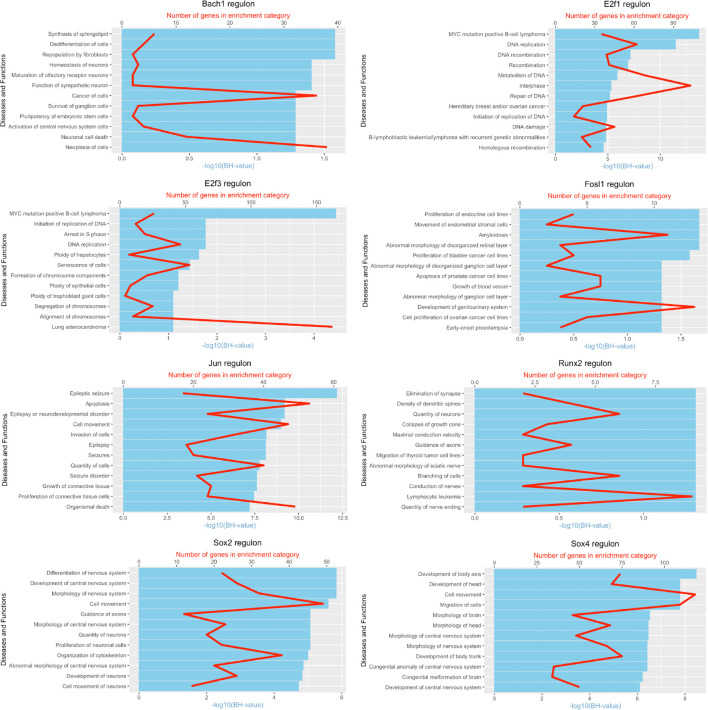
Pathway and functional enrichment analysis of Bach1, E2f1, E2f3, Fosl1, Jun, Runx2, Sox2, and Sox4 in Schwann cells at day 3 post-injury.

**FIGURE 11:**
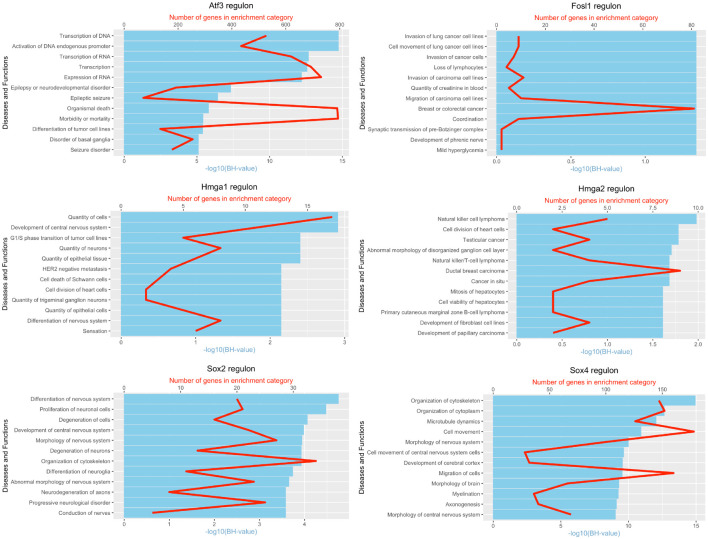
Pathway and functional enrichment analysis of Atf3, Fosl1, Hmga1, Hmga2, Sox2, and Sox4 in Schwann cells at day 9 post-injury.

It is well known that Schwann cells are the most important cell type in the distal nerve to promote peripheral nerve regeneration (Jessen and Mirsky, 2016), and our recent scRNA-seq data analysis showed that Schwann cells in the distal nerve have the highest amount of gene expression changes in response to injury (Chen et al., 2021). Here we focused on the analysis of the transcription factor network and functional analysis in Schwann cells on the scRNA-seq data using an R package SCENIC. Following SCENIC analysis, we selected TFs that belong to above 55 identified TFs for further network and functional analysis. This analysis has identified eight high confidence TFs (Bach1, E2f1, E2f3, Fosl1, Jun, Runx2, Sox2, and Sox4) in the day 3 scRNA-seq data set (Figures 6, 7), and identified six high confidence TFs (Atf3, Fosl1, Hmga1, Hmga2, Sox2, and Sox4) in the day 9 scRNA-seq data set (Figures 8, 9). Fosl1, Sox2, and Sox4 have been identified as high confidence TFs in Schwann cells at both day 3 and day 9 data sets. Next, we created transcription factor regulatory networks using SCENIC for Bach1, E2f1, E2f3, Fosl1, Jun, Runx2, Sox2, and Sox4 in Schwann cells at day 3 post-injury (Supplementary Figure 1), and created transcription factor regulatory networks for Atf3, Fosl1, Hmga1, Hmga2, Sox2, and Sox4 in Schwann cells at day 9 post-injury (Supplementary Figure 2). Finally, we performed gene enrichment analysis on the scRNA-seq data using Ingenuity software to look at the functional role of target genes in Schwann cells for each TF, and the most significant 12 functions were plotted (Figures 10, 11). This analysis showed that the up-regulation of Atf3, Bach1, E2f1, E2f3, Fosl1, Hmga1, Hmga2, Jun, Runx2, Sox2, and Sox4 in Schwann cells could regulate gene expression in Schwann cells that are important to control cell survival, proliferation, migration, myelination, and axonal guidance during peripheral nerve regeneration.

## Discussion

In recent years, scRNA-seq technologies have been widely applied to different tissues of various species to reveal cell types and meaningful gene expression profiles (Hwang et al., 2018; Chen et al., 2019). However, this technology has only recently been applied onto healthy and injured mouse sciatic nerve to study the cell types present and their gene expression profiles (Carr et al., 2019; Kalinski et al., 2020; Toma et al., 2020; Wolbert et al., 2020). So far, only two scRNA-seq data sets are publicly available for this analysis that have studied cell types and gene expression profiles in the distal nerve stump at day 3 and day 9 following mouse sciatic nerve transection injury (Carr et al., 2019; Toma et al., 2020). A number of scRNA-seq technologies such as Drop-seq and Smart-seq2 have been developed and these methods possess their own unique features with distinct advantages and disadvantages (Hwang et al., 2018; Chen et al., 2019). Due to the complexity of single cell preparation procedures and the low amount of mRNA for some genes in individual cells, studies have shown that scRNA-seq data are often “noisier” and more complex than microarray data and bulk RNA-seq data (Hwang et al., 2018; Chen et al., 2019). Both data sets that we used in this study were generated by Drop-seq technology (Carr et al., 2019; Toma et al., 2020). The process of tissue dissociation and single cell separation of Drop-seq could not only alter the level of gene expression but could also induce gene expression not found *in vivo* (Vanlandewijck et al., 2018). Therefore, we first analysed TF upregulation in the mouse distal sciatic nerve from published microarray data sets.

Searching published microarray data sets revealed that there are three data sets available for analysis at day 3 post-injury but there is no data set available at day 9 post-injury. We therefore chose day 7 post-injury for analysis, which is a time point that not only is close to day 9 post-injury but also has four data sets available for analysis. Analysing four microarray data sets identified 56 TFs in the mouse distal sciatic nerve that have at least twofold upregulation at day 3 and day 7 post-injury. Although we identified Myod1 up-regulation in our microarray data analysis, our scRNA-seq data analysis showed that Myod1 is not expressed in the distal nerve following mouse sciatic nerve injury at either day 3 or day 9 post-injury. Furthermore, Myod1 mRNA was undetectable in the bulk mRNA sequencing data (Figure 1). Thus, only 55 TFs have been further examined for their expression in different cell types of the distal nerve stump at day 3 and day 9 post-injury.

Our microarray analysis showed that Irf7 was only upregulated at day 3 post-injury, and the bulk mRNA analysis indicated that the Irf7 mRNA level at day 5 post-injury is significantly lower than its mRNA level in the intact nerve (Figure 1C). scRNA-seq analysis revealed that Irf7 is highly expressed in recruited B cells at day 3 post-injury (Figure 2), but at day 9 post-injury B cells in the distal nerve express little Irf7 (Figure 3). IRF7 is a lymphoid-specific factor, which is constitutively expressed in the cytoplasm in B cells (Ning et al., 2011). This indicates that the Irf7 upregulation in the distal nerve at day 3 post-injury may result from B cell recruitment. The distal nerve environment actually appears to downregulate Irf7 in B cells following recruitment. Thus, the upregulation of some TFs in the distal nerve stump may result from immune cell recruitment.

So far, among the 55 identified TFs, only the function of Jun, Sox2, and Fos in Schwann cells have been studied during peripheral nerve regeneration (Arthur-Farraj et al., 2012; Fontana et al., 2012; Roberts et al., 2017; Ko et al., 2018; Dun et al., 2019). The function of other identified TFs in peripheral nerve regeneration remains to be characterised. Jun is the best-studied TF for its function in Schwann cells during peripheral nerve regeneration and plays important roles in Schwann cell reprogramming, myelin clearance and the production of Schwann cell derived growth factors (Arthur-Farraj et al., 2012; Fontana et al., 2012). Our analysis revealed that Jun is highly expressed in Schwann cells, endoneurial fibroblasts and endothelial cells at day 3 following injury, while at day 9 following injury, it has its highest expression in mast cells. Thus, the function of Jun in endoneurial fibroblasts, endothelial cells and mast cells remains to be characterised. Our analysis also revealed that Junb has the highest expression in neutrophils of the injured peripheral nerves and a function for Junb in neutrophils during peripheral nerve regeneration remains to be elucidated. A previous study showed that the expression of Fos was increased in Schwann cells following peripheral nerve injury (Ko et al., 2018). Ko et al. investigated the relationship between hepatocyte growth factor (HGF) and Fos in Schwann cells. They showed that HGF increased Fos expression in cultured rat primary Schwann cells, and Fos not only regulates Schwann cell migration but also induced glial cell-derived neurotrophic factor (GDNF), leukemia inhibitory factor (LIF) and Myc expression in Schwann cells (Ko et al., 2018). Our analysis in this study showed that Fos has its highest expression in endothelial cells and immune cells; therefore, it will be interesting to investigate the function of Fos in endothelial cells and immune cells during peripheral nerve regeneration.

Our analysis revealed that Sox2 is exclusively upregulated in Schwann cells of the distal nerve stump. Our research group previously used both Schwann cell specific Sox2 overexpressing mice and Schwann cell specific Sox2 knockout mice and studied its role in Schwann cells during peripheral nerve regeneration (Roberts et al., 2017; Dun et al., 2019). We showed that Sox2 regulates Schwann cell proliferation, migration and re-myelination during peripheral nerve repair. Functional recovery is impaired in both Sox2 overexpressing and Sox2 knockout mice following peripheral nerve crush injury (Roberts et al., 2017; Dun et al., 2019). Our analysis revealed that Olig1 is upregulated in Schwann cells of the distal nerve after day 3 post-injury. In oligodendrocytes, Olig1 interacts with Sox10 synergistically to drive myelin basic protein transcription (Li et al., 2007). Cultured primary mouse Schwann cells express Olig1 and forskolin treatment of cultured primary mouse Schwann cells induces the expression of myelin-related genes but strongly inhibits Olig1 expression (Schmid et al., 2014). Olig1 is expressed in non-myelinating Schwann cells of postnatal peripheral nerves (Schmid et al., 2014). Thus, Olig1 appears to not be required for myelin gene expression in Schwann cells, and further investigations are required to understand the role of Olig1 in Schwann cells during development and regeneration. In the Runx TF family, Runx2 shows Schwann cell-specific upregulation at both day 3 and day 9 post-injury. Currently, we are using a Schwann cell specific Runx2 knockout mouse model and studying its function in peripheral nerve regeneration.

Using droplet-based single-nucleus RNA sequencing technology, Renthal et al. (2020) recently mapped the transcriptomes of individual mouse DRG cells across a range of nerve injury models. One of the most dramatic findings in their study was that peripheral axonal injury resulted in a profound transcriptional reprogramming of DRG neurons. They found that axonal injury induces a common transcriptional program across all neuronal subtypes. At least 24 upregulated TFs have been identified in their study, and they showed that these TFs not only upregulate injury-response genes but also downregulate neuronal subtype-specific genes that are expressed in uninjured neurons. Over half of these 24 transcription factors have been previously detected in neurons after axonal injury (Herdegen et al., 1992; Tsujino et al., 2000; Patodia and Raivich, 2012; Chandran et al., 2016; Mahar and Cavalli, 2018). Renthal et al. also observed that the activating protein (AP-1) family members such Atf3, Jun, Jund, and Fosl2 were highly associated with the motifs for the expression of injury-induced genes (Renthal et al., 2020). Jun and Atf3 are important injury-induced TFs to activate the neuronal intrinsic regeneration program following axon damage (Tsujino et al., 2000; Parsadanian et al., 2006; Hunt et al., 2012; Xu et al., 2019). Our analysis in this study revealed that the AP-1 TF family members Atf3, Jun, Junb, Fos, Fosl2, and Mafb are all highly expressed in different cell types of the distal nerve following mouse sciatic nerve injury, and these are important TFs that are all activated in response to tissue injury (Xu et al., 2019). Early studies have already shown that their expression in adult peripheral nerve is very low but is rapidly induced in the distal nerve following injury (Arthur-Farraj et al., 2012, 2017; Fontana et al., 2012; Patodia and Raivich, 2012; Blom et al., 2014; Ko et al., 2018). Thus, the AP-1 family TF members such Jun, Junb, Fos, Fosl2, and Atf3 could be important TFs regulating the distal nerve microenvironment following peripheral nerve injury, and further investigations are required to fully characterise their function in the distal nerve during regeneration.

In summary, we identified 55 TFs that are upregulated in the distal nerve following mouse sciatic nerve injury. Among all the identified TFs, Aebp1, Fos, Jun, and Junb are highly expressed in most cell types within distal injured nerve. Bach1, Fosl1, Hmga1, Hmga2, Olig1, Runx2, Sox2, Sox4, and Tfap2a show strong expression in Schwann cells. Aebp1, Glis3, and Plagl1 are highly expressed in fibroblasts. Mafb is expressed in macrophages and Nfe2 is expressed in mast cells. The remaining TFs show expression in more than one cell type of the mouse distal nerve. We validated Jun, Runx2, and Sox2 expression in Schwann cells of the mouse distal nerve stump by immunostaining using PLP-GFP mice, we also showed by immunostaining that Junb and Runx1 are expressed in GFP negative cells. However, further studies at spatial resolution (multiplexing or spatial transcriptomics) are needed to validate the expression other TFs in specific cell types of the injured peripheral nerves. We created a cell type expression map for the 55 identified TFs to reveal their possible regulatory function on gene expression in different cell types of the distal injured nerve. The map could not only be used to facilitate the understanding of cell-specific functions of key transcription factors in peripheral nerve regeneration but may guide experimental design for researchers who are interested in studying the function of individual TFs in peripheral nerve regeneration and repair.

## Data Availability Statement

The original contributions presented in the study are included in the article/Supplementary Material, further inquiries can be directed to the corresponding author.

## Ethics Statement

The animal study was reviewed and approved by Plymouth University Animal Welfare and Ethical Review Board.

## Author Contributions

XD designed the research. ML analyzed the microarray and bulk mRNA datasets. ML, MB, and XD performed scRNA-seq data analysis. QM performed immunostaining. XD and DP wrote the manuscript. All authors contributed to the article and approved the submitted version.

## Conflict of Interest

The authors declare that the research was conducted in the absence of any commercial or financial relationships that could be construed as a potential conflict of interest.

## Publisher’s Note

All claims expressed in this article are solely those of the authors and do not necessarily represent those of their affiliated organizations, or those of the publisher, the editors and the reviewers. Any product that may be evaluated in this article, or claim that may be made by its manufacturer, is not guaranteed or endorsed by the publisher.

## Footnotes

1
https://satijalab.org/seurat/

## Supplementary Material

Supplementary Figure 1 Networks for Bach1, E2f1, E2f3, Fosl1, Jun, Runx2, Sox2, Sox4, and their target genes in Schwann cells at day 3 post-injury.

Supplementary Figure 2 Networks for Atf3, Fosl1, Hmga1, Hmga2, Sox2, Sox4, and their target genes in Schwann cells at day 9 post-injury.

Supplementary Table 1 TFs with at least twofold upregulation at day 3 post-injury in microarray data sets GSE22291, GSE74087, and GSE136958.

Supplementary Table 2 TFs with at least twofold upregulation at day 7 post-injury in microarray data sets GSE22291, GSE74087, and GSE38693.

Supplementary Table 3 Expression abundance in each cell type for the identified 51 TFs at day 3 post-injury.

Supplementary Table 4 Expression abundance in each cell type for the identified 55 TFs at day 9 post-injury.

## References

[B1] Arthur-Farraj P. J. Latouche M. Wilton D. K. Quintes S. Chabrol E. Banerjee A. (2012). c-Jun reprograms Schwann cells of injured nerves to generate a repair cell essential for regeneration. *Neuron* 75 633–647. 10.1016/j.neuron.2012.06.021 22920255 PMC3657176

[B2] Arthur-Farraj P. J. Morgan C. C. Adamowicz M. Gomez-Sanchez J. A. Fazal S. V. Beucher A. (2017). Changes in the Coding and Non-coding Transcriptome and DNA Methylome that Define the Schwann Cell Repair Phenotype after Nerve Injury. *Cell Rep.* 20 2719–2734. 10.1016/j.celrep.2017.08.064 28903050 PMC5608958

[B3] Barrette B. Calvo E. Vallieres N. Lacroix S. (2010). Transcriptional profiling of the injured sciatic nerve of mice carrying the Wld(S) mutant gene: identification of genes involved in neuroprotection, neuroinflammation, and nerve regeneration. *Brain Behav. Immun.* 24 1254–1267. 10.1016/j.bbi.2010.07.249 20688153

[B4] Blom C. L. Martensson L. B. Dahlin L. B. (2014). Nerve injury-induced c-Jun activation in Schwann cells is JNK independent. *Biomed. Res. Int.* 2014:392971. 10.1155/2014/392971 24877090 PMC4022193

[B5] Carr L. Parkinson D. B. Dun X. P. (2017). Expression patterns of Slit and Robo family members in adult mouse spinal cord and peripheral nervous system. *PLoS One* 12:e0172736. 10.1371/journal.pone.0172736 28234971 PMC5325304

[B6] Carr M. J. Toma J. S. Johnston A. P. W. Steadman P. E. Yuzwa S. A. Mahmud N. (2019). Mesenchymal precursor cells in adult nerves contribute to mammalian tissue repair and regeneration. *Cell Stem Cell* 24 240–256.e9. 10.1016/j.stem.2018.10.024 30503141

[B7] Chandran V. Coppola G. Nawabi H. Omura T. Versano R. Huebner E. A. (2016). A systems-level analysis of the peripheral nerve intrinsic axonal growth program. *Neuron* 89, 956–970. 10.1016/j.neuron.2016.01.034 26898779 PMC4790095

[B8] Chen B. Banton M. C. Singh L. Parkinson D. B. Dun X. P. (2021). Single cell transcriptome data analysis defines the heterogeneity of peripheral nerve cells in homeostasis and regeneration. *Front. Cell Neurosci.* 15:624826. 10.3389/fncel.2021.624826 33828460 PMC8019921

[B9] Chen B. Carr L. Dun X. P. (2020). Dynamic expression of Slit1-3 and Robo1-2 in the mouse peripheral nervous system after injury. *Neural. Regen. Res.* 15 948–958. 10.4103/1673-5374.268930 31719262 PMC6990781

[B10] Chen G. Ning B. Shi T. (2019). Single-cell RNA-Seq technologies and related computational data analysis. *Front. Genet.* 10:317. 10.3389/fgene.2019.00317 31024627 PMC6460256

[B11] Clements M. P. Byrne E. Camarillo Guerrero L. F. Cattin A. L. Zakka L. Ashraf A. (2017). The wound microenvironment reprograms schwann cells to invasive mesenchymal-like cells to drive peripheral nerve regeneration. *Neuron* 96 98–114.e7. 10.1016/j.neuron.2017.09.008 28957681 PMC5626803

[B12] Cohen M. M. Jr. (2009). Perspectives on RUNX genes: an update. *Am. J. Med. Genet. A* 149A 2629–2646. 10.1002/ajmg.a.33021 19830829

[B13] Davies A. J. Kim H. W. Gonzalez-Cano R. Choi J. Back S. K. Roh S. E. (2019). Natural killer cells degenerate intact sensory afferents following nerve injury. *Cell* 176 716–728.e18. 10.1016/j.cell.2018.12.022 30712871 PMC6418410

[B14] Doddrell R. D. Dun X. P. Moate R. M. Jessen K. R. Mirsky R. Parkinson D. B. (2012). Regulation of Schwann cell differentiation and proliferation by the Pax-3 transcription factor. *Glia* 60 1269–1278. 10.1002/glia.22346 22532290 PMC5722199

[B15] Doddrell R. D. Dun X. P. Shivane A. Feltri M. L. Wrabetz L. Wegner M. (2013). Loss of SOX10 function contributes to the phenotype of human Merlin-null schwannoma cells. *Brain* 136 549–563. 10.1093/brain/aws353 23413263 PMC3572932

[B16] Dun X. P. Carr L. Woodley P. K. Barry R. W. Drake L. K. Mindos T. (2019). Macrophage-derived slit3 controls cell migration and axon pathfinding in the peripheral nerve bridge. *Cell Rep.* 26 1458–1472.e4. 10.1016/j.celrep.2018.12.081 30726731 PMC6367597

[B17] Evrard M. Kwok I. W. H. Chong S. Z. Teng K. W. W. Becht E. Chen J. (2018). Developmental Analysis of Bone Marrow Neutrophils Reveals Populations Specialized in Expansion, Trafficking, and Effector Functions. *Immunity* 48 364–379.e8. 10.1016/j.immuni.2018.02.002 29466759

[B18] Fontana X. Hristova M. Da Costa C. Patodia S. Thei L. Makwana M. (2012). c-Jun in Schwann cells promotes axonal regeneration and motoneuron survival via paracrine signaling. *J. Cell Biol.* 198 127–141. 10.1083/jcb.201205025 22753894 PMC3392945

[B19] Hafemeister C. Satija R. (2019). Normalization and variance stabilization of single-cell RNA-seq data using regularized negative binomial regression. *Genome Biol.* 20:296. 10.1186/s13059-019-1874-1 31870423 PMC6927181

[B20] Herdegen T. Fiallos-Estrada C. E. Schmid W. Bravo R. Zimmermann M. (1992). The transcription factors c-JUN, JUN D and CREB, but not FOS and KROX-24, are differentially regulated in axotomized neurons following transection of rat sciatic nerve. *Brain Res. Mol. Brain Res.* 14, 155–165. 10.1016/0169-328x(92)90170-g 1331648

[B21] Hunt D. Raivich G. Anderson P. N. (2012). Activating transcription factor 3 and the nervous system. *Front. Mol. Neurosci.* 5:7. 10.3389/fnmol.2012.00007 22347845 PMC3278981

[B22] Hwang B. Lee J. H. Bang D. (2018). Single-cell RNA sequencing technologies and bioinformatics pipelines. *Exp. Mol. Med.* 50 1–14. 10.1038/s12276-018-0071-8 30089861 PMC6082860

[B23] Jessen K. R. Mirsky R. (2008). Negative regulation of myelination: relevance for development, injury, and demyelinating disease. *Glia* 56 1552–1565. 10.1002/glia.20761 18803323

[B24] Jessen K. R. Mirsky R. (2016). The repair Schwann cell and its function in regenerating nerves. *J. Physiol.* 594 3521–3531. 10.1113/JP270874 26864683 PMC4929314

[B25] Jessen K. R. Mirsky R. (2019). The Success and Failure of the Schwann Cell Response to Nerve Injury. *Front. Cell Neurosci.* 13:33. 10.3389/fncel.2019.00033 30804758 PMC6378273

[B26] Kalinski A. L. Yoon C. Huffman L. D. Duncker P. C. Kohen R. Passino R. (2020). Analysis of the immune response to sciatic nerve injury identifies efferocytosis as a key mechanism of nerve debridement. *Elife* 9:e60223. 10.7554/eLife.60223 33263277 PMC7735761

[B27] Ko K. R. Lee J. Nho B. Kim S. (2018). c-Fos is necessary for HGF-mediated gene regulation and cell migration in Schwann cells. *Biochem. Biophys. Res. Commun.* 503 2855–2860. 10.1016/j.bbrc.2018.08.054 30103949

[B28] Lambert S. A. Jolma A. Campitelli L. F. Das P. K. Yin Y. Albu M. (2018). The human transcription factors. *Cell* 175 598–599. 10.1016/j.cell.2018.09.045 30290144

[B29] Li H. Lu Y. Smith H. K. Richardson W. D. (2007). Olig1 and Sox10 interact synergistically to drive myelin basic protein transcription in oligodendrocytes. *J. Neurosci.* 27 14375–14382. 10.1523/JNEUROSCI.4456-07.2007 18160645 PMC6329447

[B30] Lindborg J. A. Mack M. Zigmond R. E. (2017). Neutrophils Are Critical for Myelin Removal in a Peripheral Nerve Injury Model of Wallerian Degeneration. *J. Neurosci.* 37 10258–10277. 10.1523/JNEUROSCI.2085-17.2017 28912156 PMC5656991

[B31] Mahar M. Cavalli V. (2018). Intrinsic mechanisms of neuronal axon regeneration. *Nat. Rev. Neurosci.* 19, 323–337. 10.1038/s41583-018-0001-8 29666508 PMC5987780

[B32] Mallon B. S. Shick H. E. Kidd G. J. Macklin W. B. (2002). Proteolipid promoter activity distinguishes two populations of NG2-positive cells throughout neonatal cortical development. *J. Neurosci.* 22 876–885. 11826117 10.1523/JNEUROSCI.22-03-00876.2002 PMC6758537

[B33] Martini R. Fischer S. Lopez-Vales R. David S. (2008). Interactions between Schwann cells and macrophages in injury and inherited demyelinating disease. *Glia* 56 1566–1577. 10.1002/glia.20766 18803324

[B34] Mindos T. Dun X. P. North K. Doddrell R. D. Schulz A. Edwards P. (2017). Merlin controls the repair capacity of Schwann cells after injury by regulating Hippo/YAP activity. *J. Cell Biol.* 216 495–510. 10.1083/jcb.201606052 28137778 PMC5294779

[B35] Ning S. Pagano J. S. Barber G. N. (2011). IRF7: activation, regulation, modification and function. *Genes Immun.* 12 399–414. 10.1038/gene.2011.21 21490621 PMC4437765

[B36] Norrmen C. Figlia G. Pfistner P. Pereira J. A. Bachofner S. Suter U. (2018). mTORC1 Is transiently reactivated in injured nerves to promote c-jun elevation and schwann cell dedifferentiation. *J. Neurosci.* 38 4811–4828. 10.1523/JNEUROSCI.3619-17.2018 29695414 PMC5956991

[B37] Otalora-Otalora B. A. Henriquez B. Lopez-Kleine L. Rojas A. (2019). RUNX family: oncogenes or tumor suppressors (Review). *Oncol. Rep.* 42 3–19. 10.3892/or.2019.7149 31059069 PMC6549079

[B38] Pan B. Liu Y. Yan J. Y. Wang Y. Yao X. Zhou H. X. (2017). Gene expression analysis at multiple time-points identifies key genes for nerve regeneration. *Muscle Nerve* 55 373–383. 10.1002/mus.25225 27313142

[B39] Parkinson D. B. Bhaskaran A. Arthur-Farraj P. Noon L. A. Woodhoo A. Lloyd A. C. (2008). c-Jun is a negative regulator of myelination. *J. Cell Biol.* 181 625–637. 10.1083/jcb.200803013 18490512 PMC2386103

[B40] Parsadanian A. Pan Y. Li W. Myckatyn T. M. Brakefield D. (2006). Astrocyte-derived transgene GDNF promotes complete and long-term survival of adult facial motoneurons following avulsion and differentially regulates the expression of transcription factors of AP-1 and ATF/CREB families. *Exp. Neurol.* 200 26–37. 10.1016/j.expneurol.2006.01.014 16497298

[B41] Patodia S. Raivich G. (2012). Role of transcription factors in peripheral nerve regeneration. *Front. Mol. Neurosci.* 5:8. 10.3389/fnmol.2012.00008 22363260 PMC3277281

[B42] Quintes S. Brinkmann B. G. Ebert M. Frob F. Kungl T. Arlt F. A. (2016). Zeb2 is essential for Schwann cell differentiation, myelination and nerve repair. *Nat. Neurosci.* 19 1050–1059. 10.1038/nn.4321 27294512 PMC4964942

[B43] Renthal W. Tochitsky I. Yang L. Cheng Y. C. Li E. Kawaguchi R. (2020). Transcriptional reprogramming of distinct peripheral sensory neuron subtypes after axonal injury. *Neuron* 108 128–144.e9. 10.1016/j.neuron.2020.07.026 32810432 PMC7590250

[B44] Roberts S. L. Dun X. P. Doddrell R. D. S. Mindos T. Drake L. K. Onaitis M. W. (2017). Sox2 expression in Schwann cells inhibits myelination in vivo and induces influx of macrophages to the nerve. *Development* 144 3114–3125. 10.1242/dev.150656 28743796 PMC5611958

[B45] Schmid D. Zeis T. Schaeren-Wiemers N. (2014). Transcriptional regulation induced by cAMP elevation in mouse Schwann cells. *ASN Neuro.* 6 137–157. 10.1042/AN20130031 24641305 PMC4834722

[B46] Stierli S. Napoli I. White I. J. Cattin A. L. Cabrejos A. M. Calavia N. G. (2018). The regulation of the homeostasis and regeneration of peripheral nerve is distinct from the CNS and independent of a stem cell population. *Development* 145:dev170316. 10.1242/dev.170316 30413560 PMC6307893

[B47] Toma J. S. Karamboulas K. Carr M. J. Kolaj A. Yuzwa S. A. Mahmud N. (2020). Peripheral Nerve Single-Cell Analysis Identifies Mesenchymal Ligands that Promote Axonal Growth. *eNeuro* 7:2020. 10.1523/ENEURO.0066-20.2020 32349983 PMC7294463

[B48] Tsujino H. Kondo E. Fukuoka T. Dai Y. Tokunaga A. Miki K. (2000). Activating transcription factor 3 (ATF3) induction by axotomy in sensory and motoneurons: a novel neuronal marker of nerve injury. *Mol. Cell Neurosci.* 15 170–182. 10.1006/mcne.1999.0814 10673325

[B49] Vanlandewijck M. He L. Mae M. A. Andrae J. Ando K. Del Gaudio F. (2018). A molecular atlas of cell types and zonation in the brain vasculature. *Nature* 554 475–480. 10.1038/nature25739 29443965

[B50] Wolbert J. Li X. Heming M. Mausberg A. K. Akkermann D. Frydrychowicz C. (2020). Redefining the heterogeneity of peripheral nerve cells in health and autoimmunity. *Proc. Natl. Acad. Sci. U. S. A.* 117 9466–9476. 10.1073/pnas.1912139117 32295886 PMC7196786

[B51] Xie X. Shi Q. Wu P. Zhang X. Kambara H. Su J. (2020). Single-cell transcriptome profiling reveals neutrophil heterogeneity in homeostasis and infection. *Nat. Immunol.* 21 1119–1133. 10.1038/s41590-020-0736-z 32719519 PMC7442692

[B52] Xu C. Li Q. Efimova O. Jiang X. Petrova M. Aseyev K. (2019). Identification of Immediate Early Genes in the Nervous System of Snail Helix lucorum. *eNeuro* 6:2019. 10.1523/ENEURO.0416-18.2019 31053606 PMC6584072

[B53] Ydens E. Amann L. Asselbergh B. Scott C. L. Martens L. Sichien D. (2020). Profiling peripheral nerve macrophages reveals two macrophage subsets with distinct localization, transcriptome and response to injury. *Nat. Neurosci.* 23 676–689. 10.1038/s41593-020-0618-6 32284604 PMC7611025

[B54] Ydens E. Cauwels A. Asselbergh B. Goethals S. Peeraer L. Lornet G. (2012). Acute injury in the peripheral nervous system triggers an alternative macrophage response. *J. Neuroinflammation* 9:176. 10.1186/1742-2094-9-176 22818207 PMC3419084

[B55] Zhao Q. Eichten A. Parveen A. Adler C. Huang Y. Wang W. (2018). Single-Cell Transcriptome Analyses Reveal Endothelial Cell Heterogeneity in Tumors and Changes following Antiangiogenic Treatment. *Cancer Res.* 78 2370–2382. 10.1158/0008-5472.CAN-17-2728 29449267

[B56] Zhou Q. Liu M. Xia X. Gong T. Feng J. Liu W. (2017). A mouse tissue transcription factor atlas. *Nat. Commun.* 8:15089. 10.1038/ncomms15089 28429721 PMC5413951

[B57] Zigmond R. E. Echevarria F. D. (2019). Macrophage biology in the peripheral nervous system after injury. *Prog. Neurobiol.* 173 102–121. 10.1016/j.pneurobio.2018.12.001 30579784 PMC6340791

