# Supplementary material for: Meta-Analysis Reveals Transcription Factor Upregulation in Cells of Injured Mouse Sciatic Nerve
Source: Front Cell Neurosci. 2021 Oct 21;15:688243. doi: 10.3389/fncel.2021.688243 (PMC8567084; doi:10.3389/fncel.2021.688243)

Schwann cells

tSNE plots overlayed with regulon activity and histograms showing the distribution of regulon activity

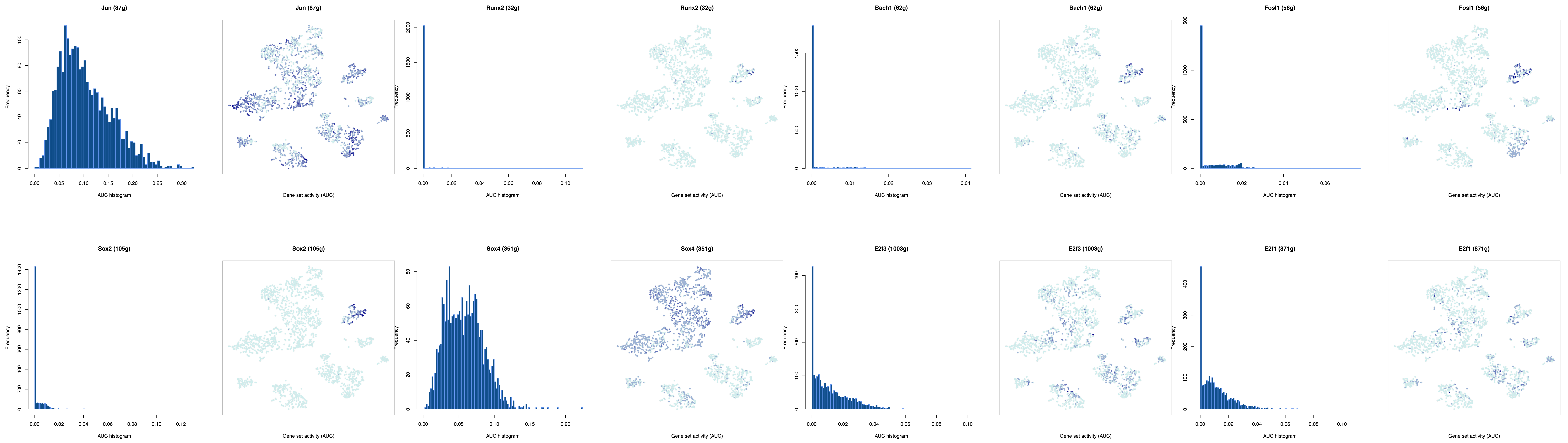

TFs described in paper: "Bach1" "Fosl1" "Hmga1" "Hmga2" "Sox2" "Sox4" "Runx2" "Cepna" "E2f1" "E2f3" "Jun" “Tcfl5"

TFs found by SCENIC and in paper: "Bach1" "Fosl1" "Sox2" "Sox4" "Runx2" "E2f1" "E2f3" "Jun"

Schwann cells

Transcription factor regulatory network and top enriched transcription factor motifs

Sequence logo of TF motif with the highest high-confidence NES (Normalised Enrichment Score). * indicates that the logo is for the highest low-confidence NES.

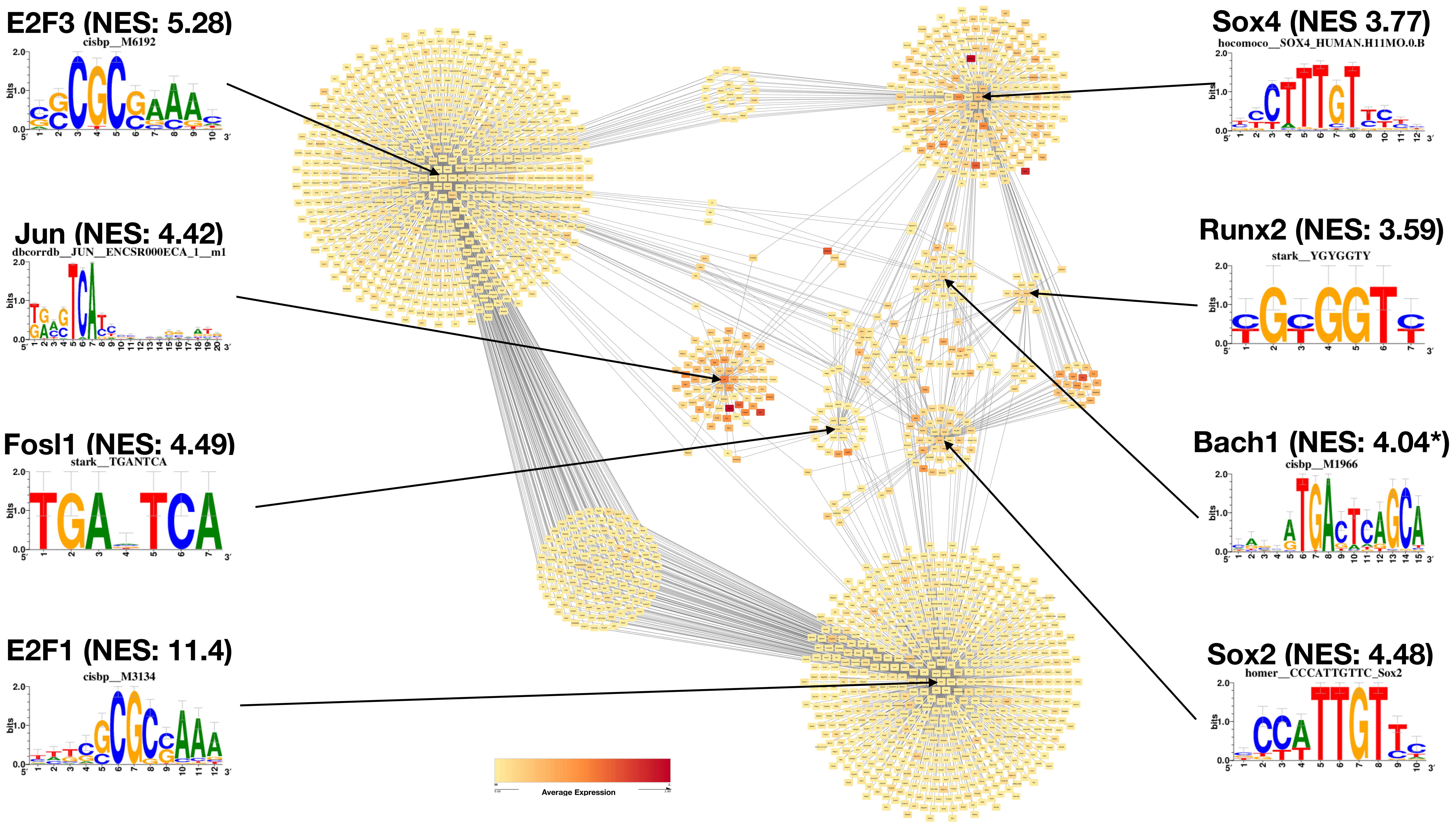

Schwann cells

Pathway and Functional enrichment analysis

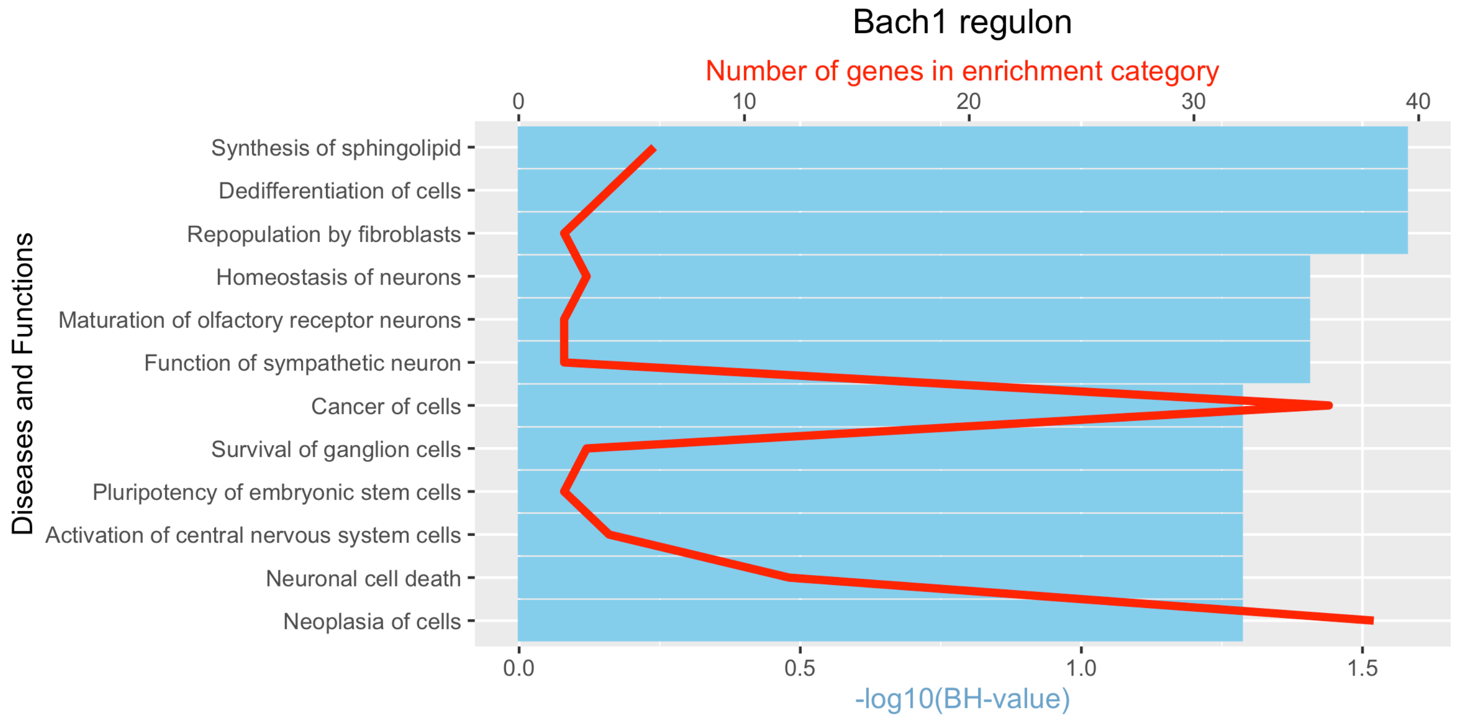

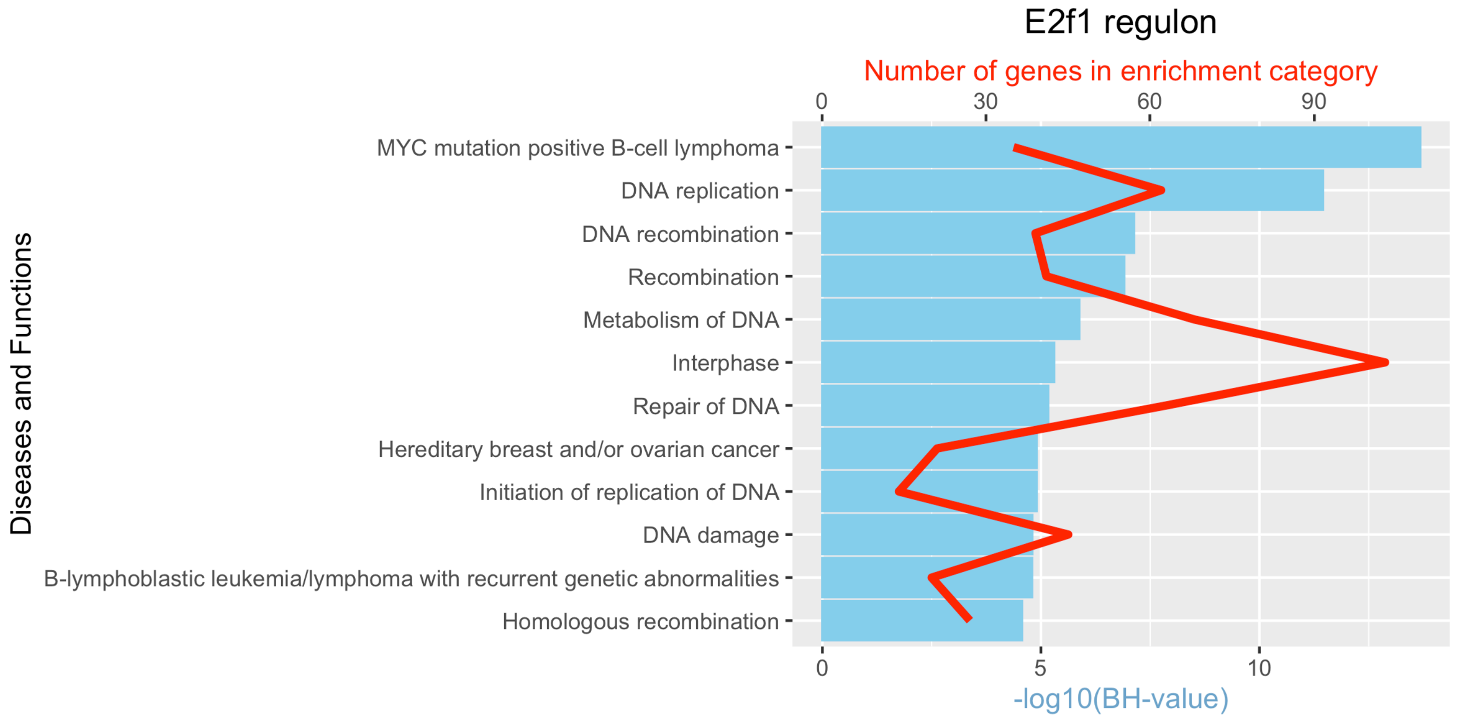

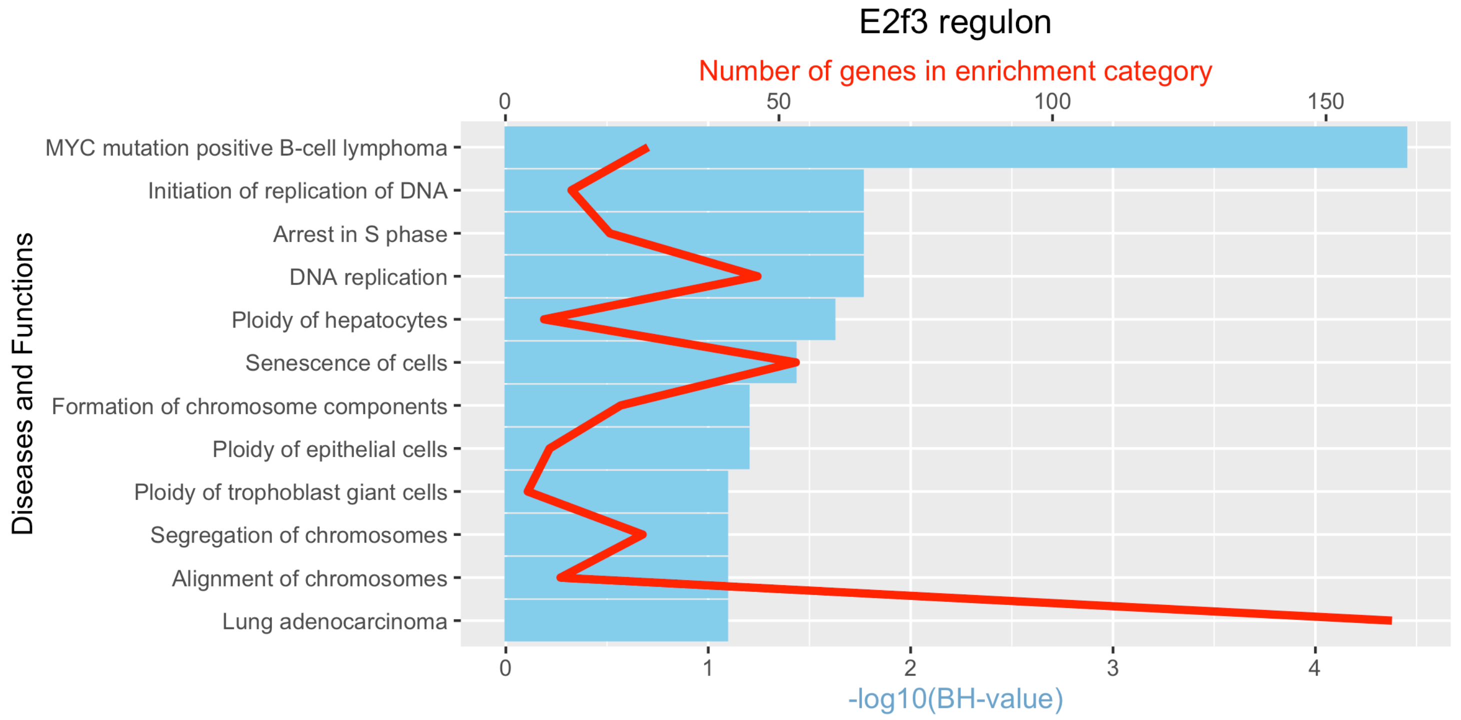

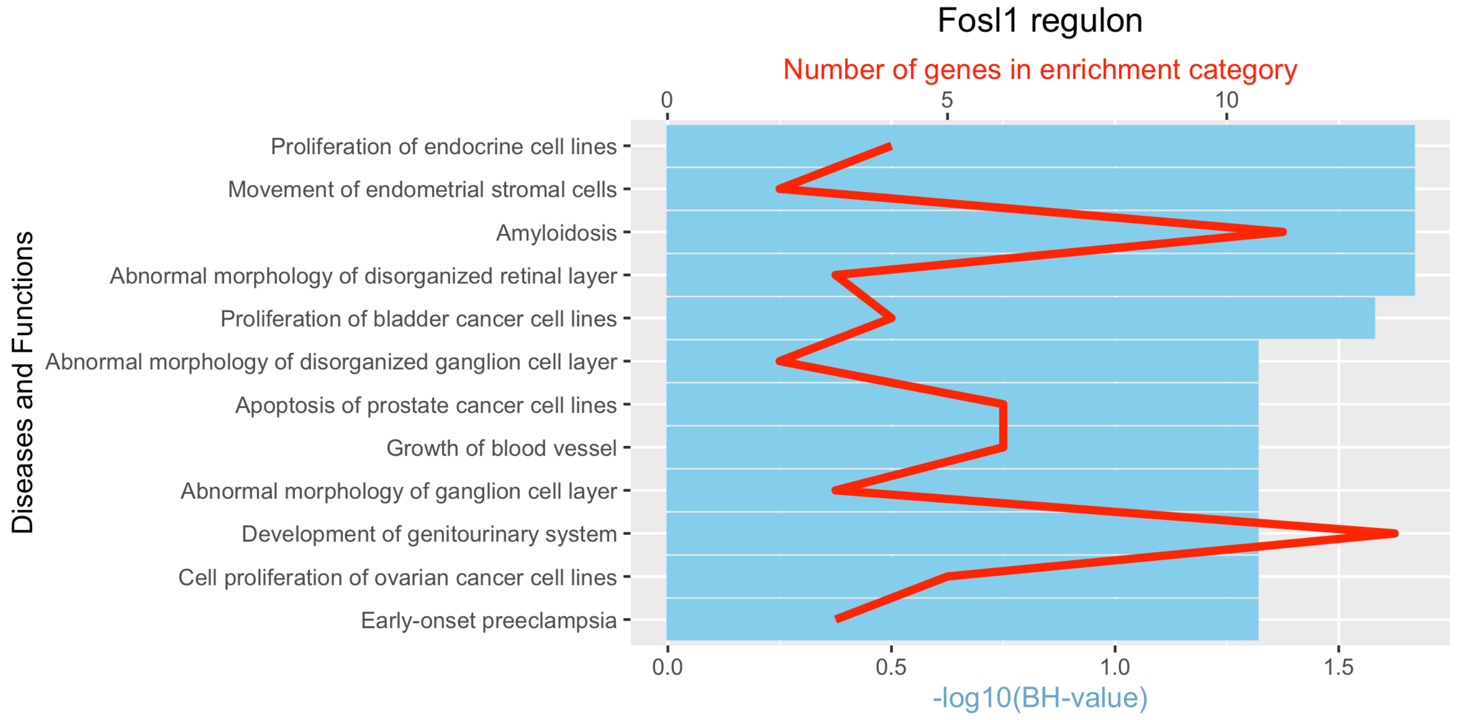

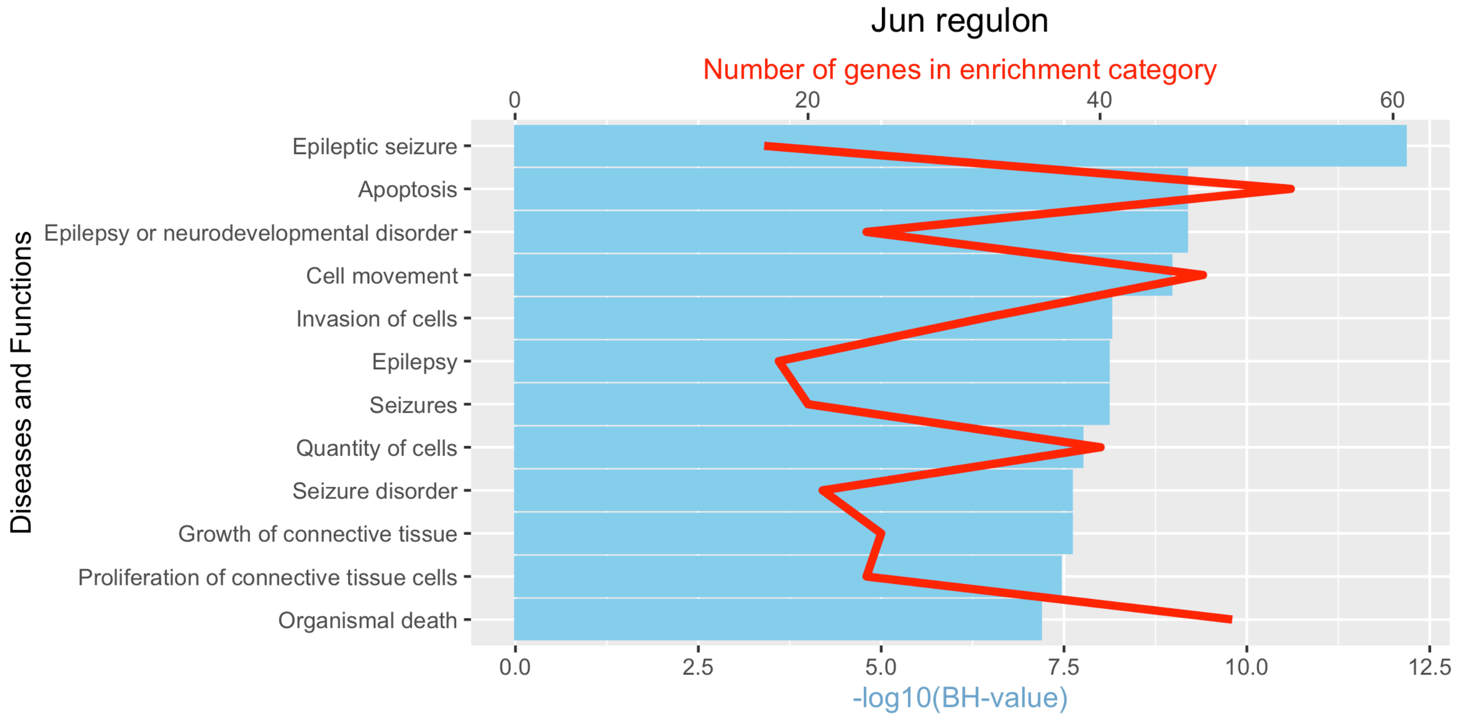

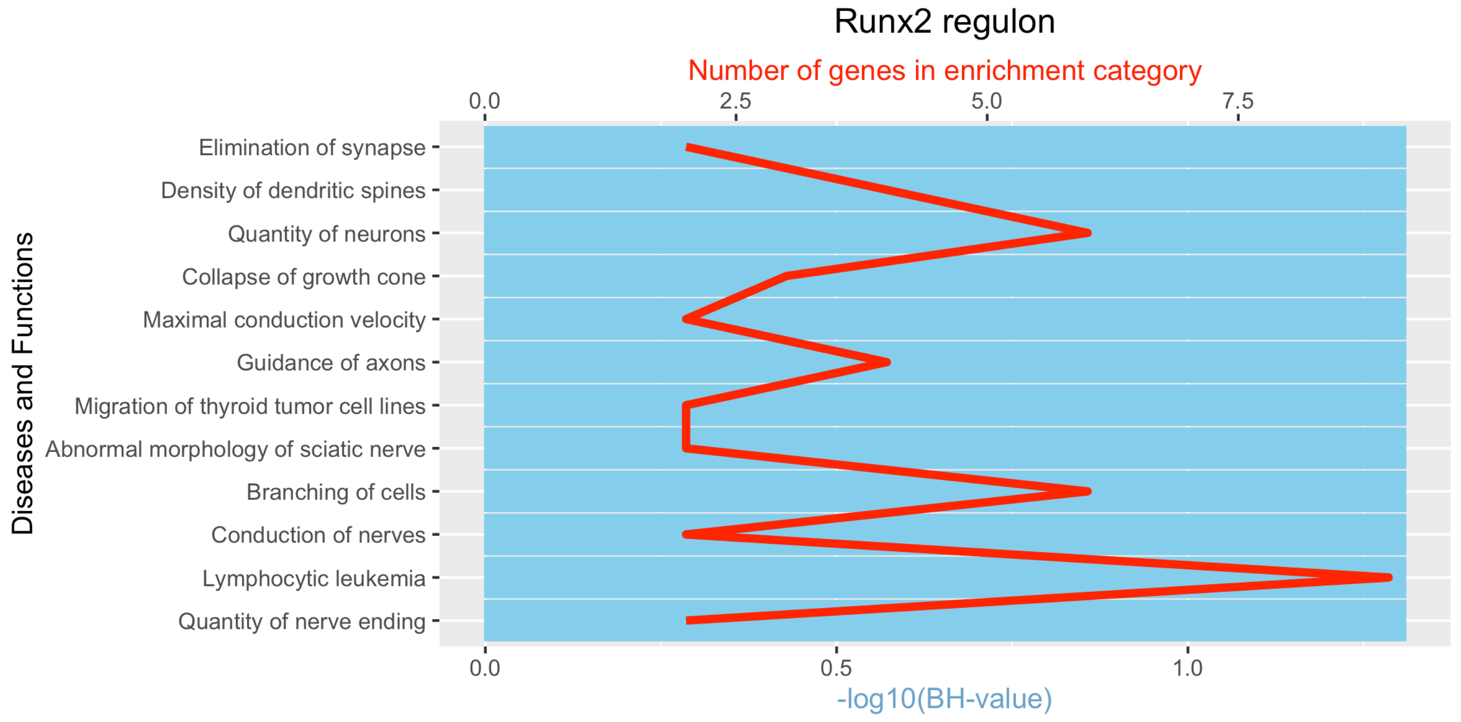

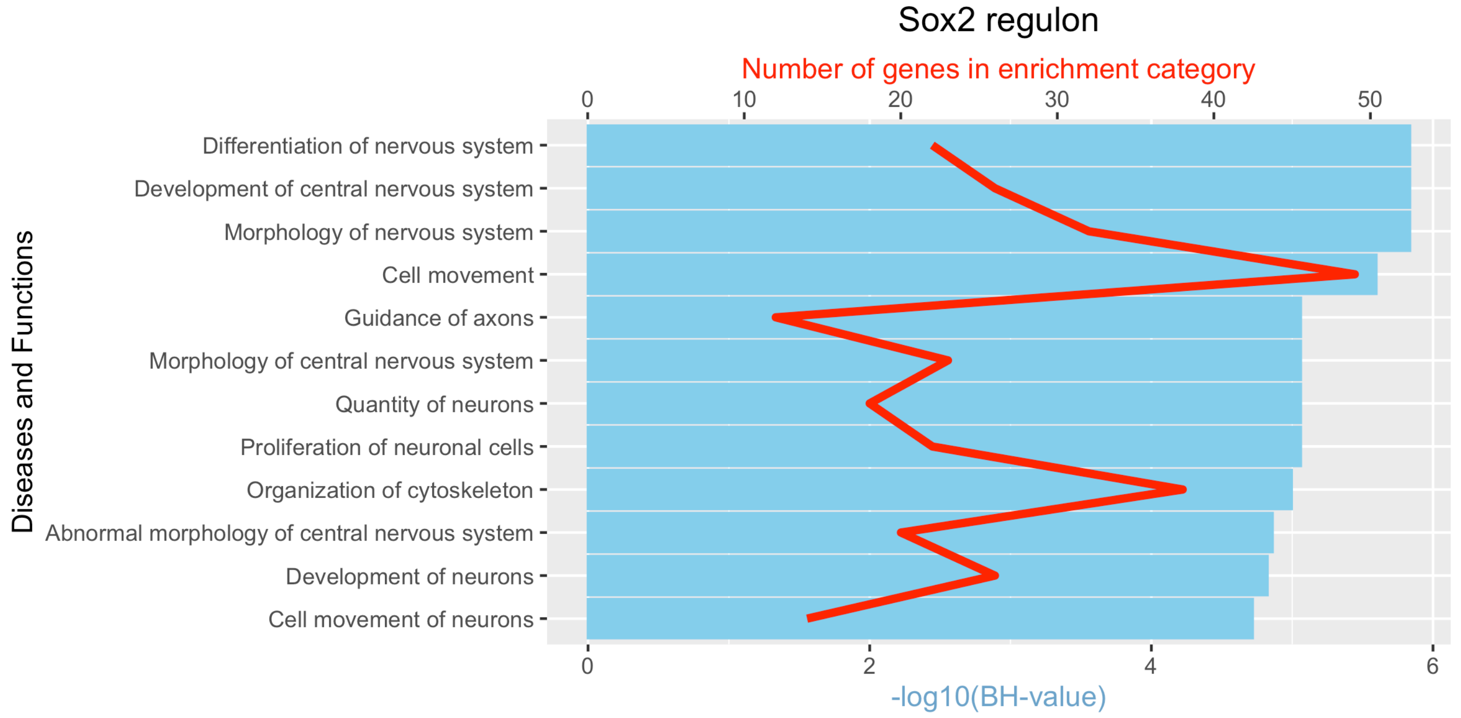

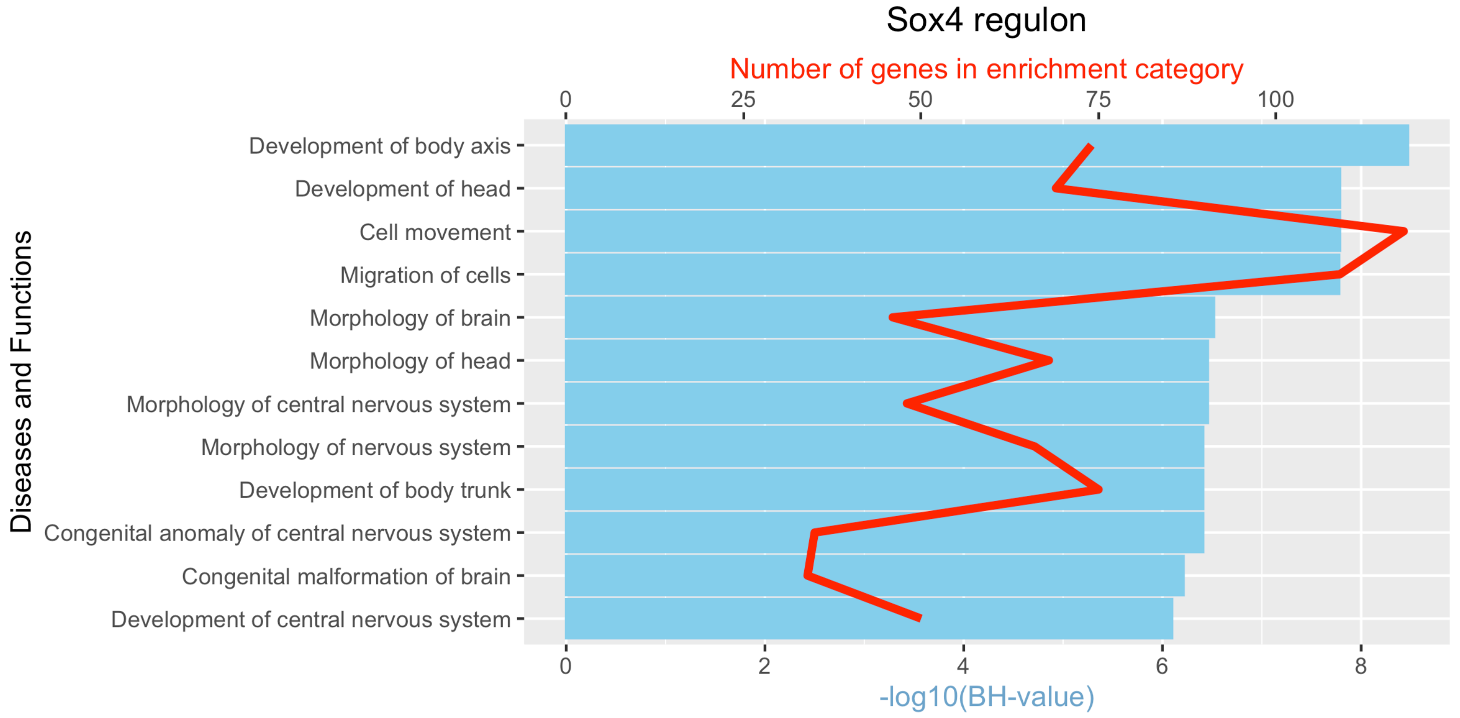

Supplement: Supplementary Figure 1 — Networks for Bach1, E2f1, E2f3, Fosl1, Jun, Runx2, Sox2, Sox4, and their target genes in Schwann cells at day 3 post-injury. [file Data_Sheet_1.PDF]
